# Integrative follicular fluid biomarkers for oocyte competence and IVF outcomes: from molecular networks to multianalyte panels

**DOI:** 10.3389/fendo.2026.1962631

**Published:** 2026-10-19

**Authors:** Chih-Ying Chen, Yu-Ting Lu, Po-Han Chang, Hsuan-Ju Chen, Ya-Wen Hsueh, Chia-Wei Chang, Hsi-Chen Hsu, Tung-Chuan Yang, Yin-Yi Chang, Hsun-Ming Chang

**Affiliations:** 1 Department of Obstetrics and Gynecology, China Medical University Hospital, Taichung, Taiwan; 2 Department of Medicine, School of Medicine, China Medical University, Taichung, Taiwan

**Keywords:** embryo developmental competence, follicular fluid, granulosa cells, *in vitro* fertilization, multianalyte biomarker panels, multi-omics integration, oocyte competence acquisition

## Abstract

Accurate assessment of oocyte developmental competence remains a major challenge in assisted reproductive technology because conventional morphological grading and ovarian-reserve markers provide limited information about the molecular state of individual follicles. Follicular fluid offers a minimally invasive window into this microenvironment, integrating systemic endocrine inputs with bidirectional oocyte–somatic cell communication, steroidogenesis, metabolic coupling, extracellular-matrix remodeling, periovulatory inflammation, redox homeostasis, mitochondrial function, and angiogenesis. This narrative review synthesizes mechanistic and clinical evidence concerning follicular-fluid biomarkers associated with oocyte maturity, fertilization, embryo development, blastocyst formation, implantation, and live birth. Particular attention is given to granulosa cell-associated signaling networks involving epidermal growth factor-like ligands, pentraxin 3, transforming growth factor-β and bone morphogenetic protein family members, activins and follistatin, glial cell line-derived neurotrophic factor, cytokines, steroid hormones, metabolic intermediates, lipids, oxidative-damage markers, and extracellular-matrix components. Available evidence indicates that individual analytes generally lack sufficient reproducibility, discrimination, and calibration for routine clinical prediction because their concentrations are influenced by patient phenotype, ovarian-stimulation regimen, follicular stage, sampling strategy, preanalytical handling, analytical platform, and outcome definition. Follicular heterogeneity and the statistical non-independence of multiple oocytes obtained from the same patient further complicate biomarker evaluation. We therefore propose a transition from single-analyte measurements toward mechanism-informed, outcome-specific multianalyte panels integrating follicular-fluid measurements with cumulus- or granulosa-cell transcriptomic, proteomic, metabolomic, and lipidomic features. Phenotype-specific models, particularly for polycystic ovary syndrome, may further improve biological interpretation. Clinical translation will require standardized individual-follicle sampling, one-to-one linkage between biomarkers and oocyte or embryo outcomes, targeted assay development, multilevel statistical modeling, independent prospective validation, and demonstration of incremental clinical utility beyond established patient and embryological variables. The ultimate goal is a parsimonious and reproducible biomarker panel that complements, rather than replaces, conventional embryological assessment and supports more individualized IVF decision-making.

## Graphical Abstract

**Figure:**
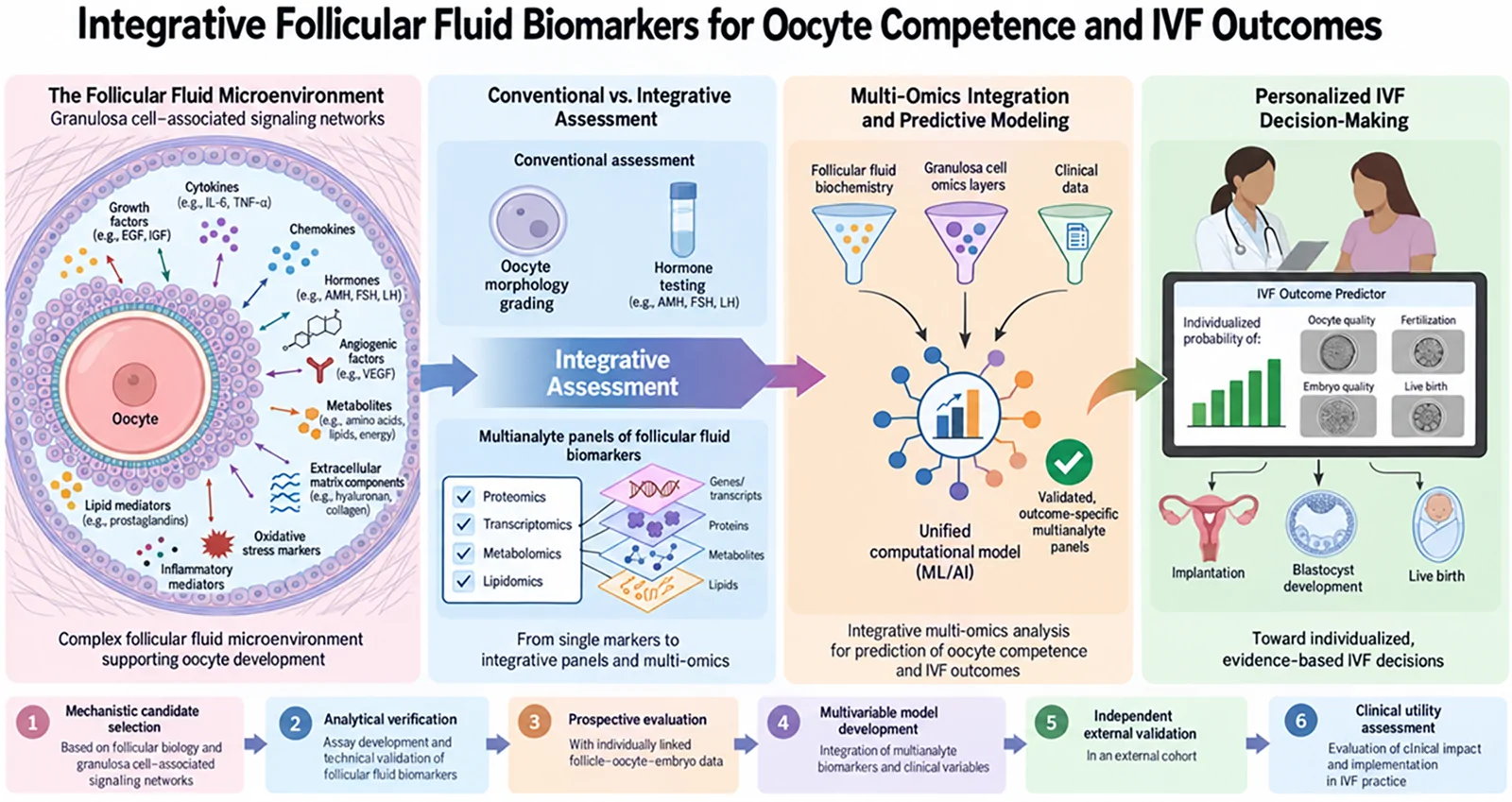
Proposed future workflow for the development and validation of follicular-fluid biomarker panels. Mechanistically selected candidates would undergo analytical verification, prospective evaluation using individually linked follicle-oocyte-embryo data, multivariable model development, and independent external validation before assessment of clinical utility. This figure represents an investigational translational framework and not an established clinical test.

## 1 Introduction

### 1.1 Clinical need for precision biomarkers in assisted reproduction

Assisted reproductive technology (ART), particularly *in vitro* fertilization (IVF), has transformed the management of infertility. Despite substantial advances in ovarian stimulation, laboratory procedures, embryo culture, and cryopreservation, accurately predicting the developmental competence of individual oocytes and embryos remains challenging. Oocyte and embryo selection continues to rely largely on morphological assessment, which provides valuable information about structural development but only incompletely reflects underlying molecular competence (1). Similarly, anti-Müllerian hormone (AMH) and antral follicle count are useful indicators of ovarian reserve and the expected response to stimulation, but they do not reliably predict the quality or reproductive potential of an individual oocyte (2).

These limitations have stimulated considerable interest in follicular fluid (FF) as a source of biomarkers for oocyte competence and IVF outcomes. Follicular development requires coordinated bidirectional communication among the oocyte, cumulus and mural granulosa cells, and theca cells (3). FF constitutes the immediate microenvironment in which these interactions occur and contains hormones, growth factors, cytokines, metabolites, lipids, extracellular vesicles, and extracellular matrix (ECM)-related molecules derived from both circulating plasma and follicular cells (4). Its composition therefore reflects the cumulative endocrine, paracrine, autocrine, metabolic, inflammatory, and oxidative processes that shape oocyte maturation.

Importantly, FF is routinely aspirated during oocyte retrieval and can therefore be obtained without an additional invasive procedure. When individual follicles can be reliably linked to their corresponding oocytes and embryos, FF analysis offers a unique opportunity to investigate follicle-specific determinants of developmental competence. Accordingly, FF-based biomarker profiling may complement conventional morphological assessment and facilitate more precise prediction of fertilization, embryo development, implantation, and pregnancy outcomes.

### 1.2 Why is a single biomarker insufficient

Folliculogenesis is governed by a highly interconnected and dynamically regulated molecular network. Growth factors, cytokines, steroid hormones, gonadotropins, metabolites, extracellular vesicles, and ECM components interact across multiple cell types and developmental stages (5). Consequently, the concentration of a single molecule is unlikely to capture the biological state of the entire follicular unit or consistently predict the developmental potential of its enclosed oocyte.

Several features of follicular biology explain the limited performance of single-analyte biomarkers. First, many signaling molecules are pleiotropic and exert context-dependent effects. Activin A, for example, participates in the regulation of granulosa-cell differentiation and steroidogenesis and may also influence cumulus–oocyte complex expansion through the regulation of matrix-associated genes, including hyaluronan synthase 2 (HAS2) and pentraxin 3 (6, 7). Thus, the biological significance of its FF concentration cannot be interpreted independently of related ligands, receptors, binding proteins, and downstream signaling activity.

Second, substantial functional redundancy exists within follicular signaling networks. Multiple members of the transforming growth factor-β (TGF-β) superfamily, including activins, TGF-β isoforms, and bone morphogenetic proteins (BMPs), may activate overlapping SMAD-dependent or alternative signaling pathways (3). These pathways can converge on common processes such as steroidogenesis, granulosa-cell differentiation, ECM remodeling, and cumulus expansion (3). This redundancy enhances the robustness of follicular development but also means that alterations in one molecule may be compensated for by changes elsewhere in the network. Accordingly, apparently normal concentrations of a single analyte do not necessarily indicate normal pathway activity, whereas an isolated abnormal value may not translate into impaired function (8).

Third, follicular signaling is both temporally and spatially organized. The transcriptomic and secretory profiles of the oocyte and surrounding somatic cells change markedly during follicular recruitment, selection, maturation, ovulation, and luteinization (refers to the LH- or hCG-induced differentiation of granulosa and theca cells into progesterone-producing luteal cells, accompanied by changes in steroidogenesis, gene expression, vascularization, and extracellular-matrix remodeling). In parallel, mural granulosa cells, cumulus cells, and theca cells occupy distinct microanatomical compartments and perform specialized functions. Some ligands, receptors, and downstream effectors are restricted to particular cell populations or developmental stages (1). FF concentrations measured at oocyte retrieval therefore represent a time-specific composite signal influenced by follicle size, maturation status, ovarian stimulation, trigger regimen, and the interval between triggering and aspiration.

These considerations support a conceptual shift from isolated biomarkers toward integrated, multi-analyte panels. Rather than relying on a single concentration threshold, biomarker combinations may better capture coordinated pathway activity, interactions among complementary biological processes, and the overall state of the follicular microenvironment. Mechanistically informed panels incorporating markers of endocrine signaling, granulosa-cell function, metabolism, inflammation, oxidative stress, angiogenesis, and ECM remodeling may therefore provide greater biological relevance and predictive stability than empirically selected individual analytes.

### 1.3 Scope and aims of this review

This review critically evaluates the FF microenvironment as a source of combined biomarkers associated with oocyte quality and IVF outcomes. Specifically, it aims to: (i) classify the principal molecular components of FF and summarize their associations with oocyte maturation, fertilization, embryo development, implantation, and pregnancy; (ii) integrate evidence from FF studies with mechanistic findings from human granulosa and cumulus cells; and (iii) propose biologically coherent, pathway-based biomarker combinations rather than isolated candidate markers; and (iv) examine the analytical, methodological, and clinical requirements for their translation into ART practice.

Particular attention is given to preanalytical variability, follicle-to-oocyte traceability, assay standardization, confounding by patient characteristics and treatment protocols, model validation, and the incremental value of biomarker panels beyond established clinical and embryological predictors. Where current evidence is insufficient to support specific biomarker combinations or causal interpretations, these limitations and priorities for future investigation are explicitly identified.

## 2 Biological basis: follicular fluid as a determinant of oocyte competence

### 2.1 Cellular and systemic origins of FF components

FF is not a static ultrafiltrate but a dynamic biological compartment whose composition evolves throughout follicular growth and periovulatory maturation. It is formed through the selective transfer of circulating plasma constituents across the vascularized theca layer and blood–follicle barrier, together with secretory products and metabolites generated locally by the oocyte, granulosa cells, cumulus cells, and theca cells (3). Consequently, FF integrates systemic endocrine and metabolic signals with follicle-specific paracrine and autocrine activity, thereby providing both a functional microenvironment for the developing oocyte and a molecular record of the follicle’s physiological state.

The blood–follicle barrier is selectively permeable rather than freely diffusible. Its permeability is influenced by molecular size, charge, protein binding, lipophilicity, and follicular vascular dynamics. Small circulating molecules, including ions, glucose, amino acids, and some steroid hormones, can enter the follicular compartment relatively readily, whereas the passage of large plasma proteins and lipoprotein particles is more restricted. Albumin and high-density lipoprotein (HDL) are prominent plasma-derived constituents of human FF, while larger lipoproteins are largely excluded (9). HDL serves as an important carrier of cholesterol and other bioactive lipids within the follicle. Cholesterol may subsequently be taken up by steroidogenic cells through receptor-mediated mechanisms, including scavenger receptor class B type 1, rather than entering the follicle exclusively through unrestricted passive diffusion (10) (**Figure 1**).

**Figure 1:**
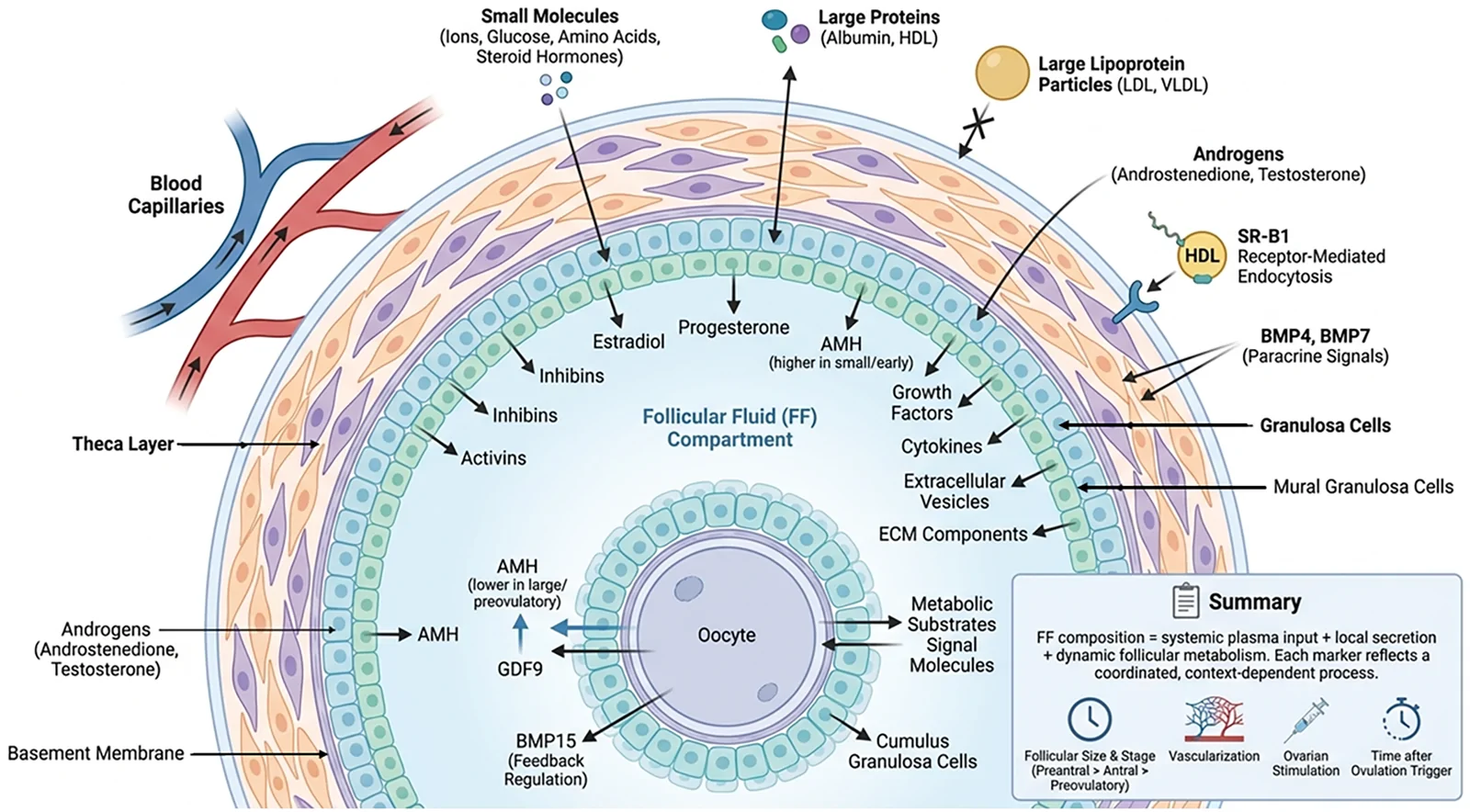
Cellular and systemic determinants of the follicular-fluid microenvironment. Follicular fluid (FF) reflects the combined effects of selective transfer from the circulation and local secretion and metabolism by theca cells, mural granulosa cells, cumulus cells, and the oocyte. Small circulating molecules readily enter the follicle, whereas the passage of larger proteins and lipoproteins is selectively regulated by the blood–follicle barrier. HDL-derived cholesterol supports steroidogenesis, whereas the entry of LDL and VLDL particles is relatively restricted. Theca cells produce androgens and paracrine factors, including BMP4 and BMP7, while granulosa cells convert androgens to estradiol and secrete steroid hormones, AMH, inhibins, activins, cytokines, growth factors, extracellular vesicles, and ECM components. Bidirectional communication between the oocyte and cumulus cells is mediated by metabolic exchange and oocyte-derived factors, including GDF9 and BMP15. Arrows indicate transport, secretion, or paracrine communication; the crossed arrow indicates restricted passage. FF biomarker concentrations therefore reflect integrated systemic and local pathway activity and should be interpreted according to follicular size and developmental stage, vascularization, ovarian-stimulation protocol, ovulation-trigger regimen, and the trigger-to-retrieval interval. AMH, anti-Müllerian hormone; BMP, bone morphogenetic protein; BMP15, bone morphogenetic protein 15; ECM, extracellular matrix; GDF9, growth differentiation factor 9; HDL, high-density lipoprotein; LDL, low-density lipoprotein; VLDL, very-low-density lipoprotein; AMH, anti-Müllerian hormone; BMP, bone morphogenetic protein; ECM, extracellular matrix; GDF9, growth differentiation factor 9; HDL, high-density lipoprotein; LDL, low-density lipoprotein; SR-B1, scavenger receptor class B type 1; VLDL, very-low-density lipoprotein.

Local somatic cells make distinct but complementary contributions to the FF proteome, metabolome, and hormonal milieu. Granulosa cells synthesize estradiol, progesterone, inhibins, activins, AMH, and numerous growth factors, cytokines, chemokines, ECM components, and extracellular vesicles (11–13). The relative contribution of these molecules varies with follicular stage and granulosa-cell differentiation. For example, AMH is produced predominantly by granulosa cells of preantral and small antral follicles, declines after follicular selection, and is expressed only minimally in the mural granulosa cells of large preovulatory follicles (12). Thus, the concentration of AMH in FF from follicles aspirated during IVF should be interpreted as a marker of developmental stage and local granulosa-cell activity rather than as a simple reflection of circulating AMH (**Figure 1**).

Theca cells constitute another major source of intrafollicular signals. Under luteinizing hormone (LH) stimulation, theca interna cells synthesize androgens, predominantly androstenedione and testosterone. These steroids traverse the follicular basement membrane and are converted to estrogens by follicle-stimulating hormone (FSH)-induced aromatase in granulosa cells, consistent with the two-cell, two-gonadotropin model of ovarian steroidogenesis (14). Theca cells also produce paracrine regulators, including BMP4 and BMP7, which act on adjacent granulosa cells to modulate gonadotropin responsiveness, steroidogenesis, proliferation, and luteinization (5) (**Figure 1**). However, the cellular localization and biological effects of individual BMPs may vary according to species and follicular stage; extrapolation from animal models to the human ovary should therefore be made cautiously.

The oocyte is likewise an active regulator of its microenvironment. Its best-characterized secretory products include growth differentiation factor 9 (GDF9) and BMP15, two members of the TGF-β superfamily that act on cumulus and granulosa cells (11). These oocyte-secreted factors regulate somatic-cell proliferation and differentiation, metabolism, ECM formation, cumulus expansion, and transzonal-projection-dependent communication. In turn, cumulus cells supply the oocyte with metabolic substrates, second messengers, and regulatory molecules required for meiotic and cytoplasmic maturation (11). This bidirectional communication creates a regulatory feedback system through which the oocyte influences the somatic-cell phenotype that supports its own developmental competence (**Figure 1**).

Accordingly, the abundance of a molecule in FF cannot automatically be attributed to a single cellular source or transport mechanism. Its measured concentration reflects the balance among local synthesis, plasma transfer, cellular uptake, extracellular binding, enzymatic processing, degradation, and clearance. This balance is further modified by follicular size, vascularization, oocyte maturation, granulosa-cell luteinization, ovarian stimulation, the ovulation-trigger regimen, and the interval between triggering and follicular aspiration (11, 15). FF collected during IVF should therefore be regarded as a stage-specific and treatment-influenced biospecimen. These biological features reinforce the need to interpret candidate FF biomarkers within coordinated pathways and well-defined clinical contexts rather than as isolated analytes (**Table 1**).

**Table 1:** Mechanism-informed follicular fluid and follicular cell biomarkers relevant to oocyte competence.

Biological module	Candidate biomarkers	Principal biological interpretation	Reported association with IVF outcomes	Evidence status and limitations
Periovulatory EGF signaling	FF AREG, EREG, BTC and PGE2; granulosa-cell PTGS2	Reflects LH/hCG-induced EGFR-ERK1/2 signaling, prostaglandin synthesis, cumulus expansion and ovulatory activation	AREG is abundant after hCG exposure; EGF-like ligands induce PTX3 and PGE2 production in human granulosa cells (16)	Strong mechanistic evidence, but clinical associations are inconsistent. AREG is the most analytically suitable ligand; EREG is detected less consistently, and BTC may be below assay detection limits
Cumulus-matrix organization	FF PTX3 and hyaluronan; cumulus-cell PTX3, HAS2, VCAN and TNFAIP6	Represents synthesis, organization and stabilization of the hyaluronan-rich cumulus extracellular matrix	PTX3 has been associated with oocyte maturity and competence in some cohorts, including PCOS; results have not been consistently replicated (6, 7, 17, 18)	PTX3 is a mechanistically integrative candidate rather than a specific marker of inflammation or oocyte quality. FF protein and cumulus-cell transcript measurements are not interchangeable
TGF-beta/BMP signaling	FF BMP4, BMP6, BMP7, TGF-beta1 and GDF8; corresponding SMAD-related cellular readouts	Provides regulatory context for cumulus expansion, steroidogenesis, gap-junction communication, extracellular-matrix remodeling and luteinization	BMP4, BMP6, BMP7, TGF-beta1 and GDF8 suppress PTX3 through distinct pathways; BMP6 and GDF8 alterations have been reported in PCOS (18–22)	Predominantly mechanistic evidence from granulosa-lutein cell models. Ligand concentration does not necessarily represent receptor occupancy or downstream pathway activity
Activin-follistatin balance	FF activin A, B and AB; follistatin and follistatin-like 3	Reflects net activin bioactivity affecting StAR, progesterone, PTX3, HAS2, versican, CTGF and LOX	Altered TGF-beta-family profiles occur in PCOS and with follicular development; no validated activin-to-follistatin threshold predicts IVF outcomes (6, 7, 14)	Biologically plausible pathway module, but clinical predictive evidence is insufficient and varies by activin isoform and follicle size
Steroidogenesis and follicular differentiation	FF estradiol, progesterone, androgens and AMH; P4/E2 ratio	Represents integrated theca-granulosa steroidogenesis, aromatase activity, luteinization and local restraint of FSH responsiveness	Individual-follicle AMH and progesterone have been associated with fertilization, embryo development, blastocyst formation and live birth in selected cohorts (23–26)	More direct clinical evidence than for many growth factors, but proposed thresholds vary with follicle size, stimulation protocol, trigger timing and assay platform
Optional exploratory cumulus-cell module GDNF-miR-145-5p axis	Cumulus-cell miR-145-5p, GFRA1, RET and EGFR; FF GDNF only as a contextual analyte	Exploratory cell-associated signal reflecting GDNF-responsive cumulus-cell survival and support of oocyte nuclear maturation	A single human IVM study reported that GDNF reduced cumulus-cell miR-145-5p and apoptosis and increased MII maturation. It did not significantly improve fertilization, cleavage or blastocyst formation (27)	**Exploratory evidence from one non-replicated human IVM study. This axis is not a core FF biomarker component and requires independent replication, analytical validation and confirmation in prospectively matched cumulus-oocyte datasets**
Extracellular-matrix turnover	FF or granulosa-cell CTGF, LOX and MMP1; granulosa-cell miR-29a and ID3	Characterizes the balance between matrix synthesis, crosslinking, degradation and periovulatory tissue remodeling	TGF-beta1 increases LOX through miR-29a suppression and decreases MMP1 through ID3; activin and BMP6 also regulate CTGF/LOX	Mechanistic evidence is substantial, but these combinations have not been prospectively validated as predictors of embryo development or luteal competence
Immune and inflammatory signaling	FF IL-6, sIL-6Ralpha, soluble gp130, PGE2 and NO metabolites	Reflects physiological periovulatory inflammation and possible pathological inflammatory or nitrosative activity	IL-6/sIL-6Ralpha induces PTGS2 and PGE2 through JAK2-STAT3; NO induces PTGS2/PGE2 through cGMP-CREB (28, 29)	IL-6 alone cannot define trans-signaling activity. Clinical associations with embryo development and pregnancy are heterogeneous and may be non-linear
Oxidative stress and antioxidant capacity	FF glutathione, total antioxidant capacity, 8-OHdG, malondialdehyde, protein carbonyls and F2-isoprostanes	Represents antioxidant buffering and oxidative damage to DNA, proteins and lipids	Higher 8-OHdG and lower glutathione or antioxidant activity have been associated with impaired fertilization and blastocyst quality in some studies (30)	Assays measure different redox domains and should not be used interchangeably. Controlled ROS production is also required for physiological ovulation
Mitochondrial function and cellular injury	FF cell-free nuclear or mitochondrial DNA, acylcarnitines and lactate/pyruvate; cumulus-cell mtDNA, ATP and membrane potential	Reflects mitochondrial abundance, metabolic stress, apoptosis and somatic-cell energetic support of the oocyte	Lower FF cell-free DNA and higher cumulus-cell mtDNA have been associated with better embryo morphology in selected cohorts (31)	Cumulus-cell measurements are not FF biomarkers. Associations are strongly affected by patient-level factors, and universal thresholds are unavailable
Metabolomic and lipidomic state	FF lysophosphatidylcholines, 3-hydroxynonanoyl carnitine, free fatty acids, amino acids and steroid metabolites	Captures downstream lipid oxidation, phospholipid remodeling, amino-acid metabolism and steroidogenesis	Distinct metabolic signatures have been reported in PCOS and in follicles associated with different developmental outcomes (32, 33)	Most findings are exploratory and disease-associated rather than outcome-predictive; targeted quantification and external validation are required
Angiogenic support	FF VEGF, CXCL12/SDF-1 and angiogenin	Reflects perifollicular vascularization, oxygen and nutrient delivery, and vascular permeability	VEGF correlates with perifollicular blood flow but not consistently with embryo quality; CXCL12 has been associated with blastocyst development (34–39)	Concentrations are influenced by age, gonadotropin exposure, hypoxia and follicular size. Angiogenic activity may have non-linear associations with competence

AREG, amphiregulin; BTC, betacellulin; CTGF, connective tissue growth factor; E2, estradiol; EGFR, epidermal growth factor receptor; EREG, epiregulin; FF, follicular fluid; GDF8, growth differentiation factor 8; GDNF, glial cell line-derived neurotrophic factor; HAS2, hyaluronan synthase 2; IL-6, interleukin 6; IVM, *in-vitro* maturation; LOX, lysyl oxidase; MII, metaphase II; MMP1, matrix metalloproteinase 1; mtDNA, mitochondrial DNA; NO, nitric oxide; 8-OHdG, 8-hydroxy-2’-deoxyguanosine; P4, progesterone; PGE2, prostaglandin E2; PTGS2, prostaglandin-endoperoxide synthase 2; PTX3, pentraxin 3; ROS, reactive oxygen species; sIL-6Ralpha, soluble interleukin-6 receptor alpha; StAR, steroidogenic acute regulatory protein; VCAN, versican; VEGF, vascular endothelial growth factor. The highlighted GDNF-miR-145-5p axis is an optional exploratory cumulus-cell module and is not part of the core follicular-fluid panel.

Bold text denotes biological module headings and highlights the exploratory status and evidence limitations of the optional GDNF–miR-145-5p cumulus-cell module. Bold formatting does not indicate statistical significance.

### 2.2 Key biological processes reflected in FF

FF contains molecular products generated by the principal processes governing follicular maturation, including oocyte–somatic-cell communication, steroidogenesis, metabolic cooperation, ECM remodeling, periovulatory inflammatory signaling, redox homeostasis, and angiogenesis. These pathways are highly interconnected and vary according to follicular stage, gonadotropin exposure, and the interval between ovulation triggering and oocyte retrieval. FF should therefore be viewed as a stage-specific composite of follicular activity rather than a direct measure of any single cellular process (**Table 2**).

**Table 2:** Mechanism-informed, outcome- and phenotype-specific research frameworks for follicular-fluid biomarkers in IVF.

Intended outcome or population	Hypothesis-driven candidate measurements	Mechanistic basis	Direct matched clinical evidence	Evidence classification	Principal next validation step
MII oocyte maturity	FF AREG, PTX3, and PGE2, with BMP4 or GDF8 as contextual pathway markers. Cumulus-cell miR-145-5p is an optional exploratory module, not a core FF component.	Human granulosa-lutein cell studies link EGF-like signaling to PTX3 and PGE2 production and BMP4/GDF8 signaling to PTX3 suppression. A single human IVM study links GDNF-mediated miR-145-5p suppression to nuclear maturation.	No prospective study has tested this complete combination in individually linked FF-oocyte datasets. The GDNF-miR-145-5p axis has not been independently replicated and was not shown to improve fertilization or blastocyst formation.	M - predominantly mechanistic; exploratory	Prospective, individually aspirated follicle-to-oocyte study with a predefined MII outcome; standardized trigger-to-retrieval interval; comparison with follicle size and conventional maturity indicators; independent replication of the miR-145-5p finding.
Normal fertilization (2PN)	FF AMH and progesterone, evaluated with selected maturity-related candidates; semen quality and IVF versus ICSI included as clinical covariates.	AMH and progesterone reflect granulosa-cell differentiation, follicular developmental status, and luteinization; the maturity-related candidates represent periovulatory and cumulus-matrix activity.	Individual-follicle studies have reported associations of FF AMH and/or progesterone with fertilization and early embryo development. No combined multianalyte panel has undergone prospective external validation.	C1/M - limited clinical associations plus mechanistic extrapolation	Prospective oocyte-matched analysis incorporating semen parameters and fertilization method; multilevel modeling of oocytes within women; assessment of incremental value beyond established clinical predictors.
Cleavage-stage embryo competence	FF AMH and progesterone with exploratory PTX3 and ECM-related candidates (TGF-beta1, CTGF, LOX and MMP1).	The proposed combination integrates endocrine support, cumulus-matrix organization and extracellular-matrix remodeling. Relationships among the ECM candidates derive largely from human granulosa-lutein cell signaling studies.	FF AMH and progesterone have limited oocyte-matched association data. The proposed ECM module has not been tested as a combined predictor in matched follicle-oocyte-embryo cohorts.	C1/M - mixed evidence; panel unvalidated	Prospective one-to-one FF-oocyte-embryo linkage; standardized cleavage-stage grading; prespecified model; demonstration of discrimination, calibration and added value beyond age and embryo morphology.
Usable or high-quality blastocyst formation	FF AMH and progesterone; oxidative-stress measures such as glutathione and 8-OHdG; cfDNA or cf-mtDNA; and selected acylcarnitine and phospholipid metabolites.	These candidates represent granulosa-cell support, redox balance, mitochondrial stress, apoptosis, and follicular metabolism. The proposed combination is pathway-based rather than empirically established.	Individual analytes or signatures have been associated with embryo or blastocyst outcomes in selected cohorts, but no complete panel has been prospectively evaluated or externally replicated with individual-follicle linkage.	C1 - early clinical association; no validated panel	Targeted analytical verification followed by prospective matched validation; distinguish blastocyst formation from blastocyst morphology; adjust for culture and laboratory conditions; perform blinded external validation.
Euploidy, implantation or live birth	No panel is presently justified. Only candidates retained after validation for maturity and blastocyst outcomes should be evaluated with age, embryo morphology or morphokinetics, and ploidy status where available.	This staged strategy tests whether FF contributes information beyond established patient and embryo predictors while avoiding premature expansion of an unvalidated signature.	Oocyte-matched FF AMH has been associated with live birth after fresh single-embryo transfer. This finding does not validate a multianalyte panel, and evidence for euploidy or implantation prediction remains insufficient.	C1 - isolated clinical association; insufficient for panel specification	Independent prospective single-embryo-transfer cohorts; prespecified live-birth endpoint; calibration and decision-curve analysis; direct comparison with models containing clinical and embryological variables alone.
PCOS-specific assessment	Exploratory modules: BMP6/PTX3; GDF8/progesterone; selected fatty acids, acylcarnitines and lysophosphatidylcholines; and IL-6 with sIL-6Ralpha.	The modules represent altered BMP/TGF-beta signaling, steroidogenesis, metabolism, and inflammatory activity in PCOS. Several relationships derive from granulosa-lutein cell experiments.	PCOS-control differences in FF or granulosa-cell measures and omics profiles have been reported, but the integrated panel has not been validated against individually matched oocyte, embryo, or live-birth outcomes.	D/M - disease-association and mechanistic evidence; exploratory for outcome prediction	Prospective outcome-linked validation stratified by PCOS phenotype, BMI, insulin resistance and hyperandrogenism; diverse populations; evaluation of population transportability and need for phenotype- or ethnicity-specific recalibration.
Other infertility phenotypes	No specific panel proposed; phenotype-directed discovery for endometriosis, diminished ovarian reserve, obesity and reproductive aging.	These disorders may affect inflammation, redox homeostasis, mitochondrial function, angiogenesis and steroidogenesis through distinct mechanisms.	Evidence is fragmented, generally based on small or heterogeneous cohorts, and does not support transferring candidate combinations or thresholds from PCOS or unselected IVF populations (33, 40).	H - hypothesis-generating only	Phenotype-specific discovery followed by analytical verification and independent prospective validation; avoid borrowing thresholds across clinically distinct populations without formal transportability analyses.

These combinations are hypothesis-generating research frameworks, not validated clinical tests. Evidence classification: M, predominantly mechanistic evidence; C1, limited observational clinical association; D, disease-association evidence; H, hypothesis-generating evidence only. None of the proposed combinations has undergone prospective, individually linked follicle-oocyte-embryo development and independent external validation. FF, follicular fluid; cfDNA, cell-free DNA; cf-mtDNA, cell-free mitochondrial DNA; ICSI, intracytoplasmic sperm injection; IVM, in-vitro maturation; MII, metaphase II; 2PN, two pronuclei. Other abbreviations are defined in the consolidated manuscript list.

#### 2.2.1 Oocyte–somatic-cell communication

Oocyte competence depends on bidirectional communication between the oocyte and its surrounding cumulus and mural granulosa cells. This communication occurs through direct intercellular contacts, particularly transzonal projections and gap junctions, as well as through secreted paracrine factors (11, 41). Gap junctions are intercellular channels assembled from connexin proteins and permit the passage of ions, metabolites, second messengers, and other molecules generally smaller than approximately 1 kDa. Connexin 37, encoded by *GJA4*, is enriched at the oocyte–cumulus-cell interface, whereas connexin 43, encoded by *GJA1*, predominantly mediates coupling among granulosa and cumulus cells (11, 42). Through these channels, somatic cells provide the oocyte with pyruvate, amino acids, nucleotides, and regulatory signals that it cannot produce efficiently. Cyclic guanosine monophosphate generated by cumulus cells also passes into the oocyte, inhibits phosphodiesterase 3A, and sustains cyclic adenosine monophosphate concentrations required to maintain meiotic arrest before the LH surge factors (11, 41).

Connexin expression and gap-junction intercellular communication are dynamically regulated by intrafollicular signals. In primary and immortalized human granulosa-lutein cells, TGF-β1 increased connexin 43 expression and intercellular communication through an ALK5–SMAD2/3–SMAD4-dependent pathway (43). These mechanistic findings should be interpreted within the limitations of human granulosa-lutein cell models. Cells collected during IVF have already been exposed to controlled ovarian stimulation and an ovulatory trigger and have therefore undergone substantial luteinization. Their phenotype may differ from that of mural granulosa and cumulus cells in unstimulated or earlier-stage follicles. Moreover, cell isolation, culture duration, serum exposure, passage, and immortalization may alter receptor expression and intracellular signaling. These models are therefore valuable for establishing biological plausibility and pathway relationships but cannot independently establish *in vivo* relevance, biomarker thresholds, or clinical predictive utility. Confirmation in physiologically relevant systems and matched follicle–oocyte studies is required. These findings support the concept that locally produced TGF-β superfamily ligands can modify somatic-cell coupling. However, most mechanistic evidence has been obtained from granulosa-lutein cells after controlled ovarian stimulation. These cells differ from non-luteinized cumulus and mural granulosa cells *in vivo*, and the findings should therefore not be interpreted as direct evidence that the corresponding FF ligand concentration quantitatively reflects gap-junction activity.

More broadly, gap-junction function represents the net effect of ligand availability, receptor expression, connexin synthesis and phosphorylation, channel trafficking, and periovulatory remodeling. Although connexin-related molecules and their regulatory ligands may contribute to a mechanistically informed biomarker panel, connexin 43 activity itself cannot currently be measured reliably from cell-free FF. Validation would require matched analyses linking FF composition, cumulus- or granulosa-cell connexin activity, and the developmental outcome of the corresponding oocyte (**Figure 2**).

**Figure 2:**
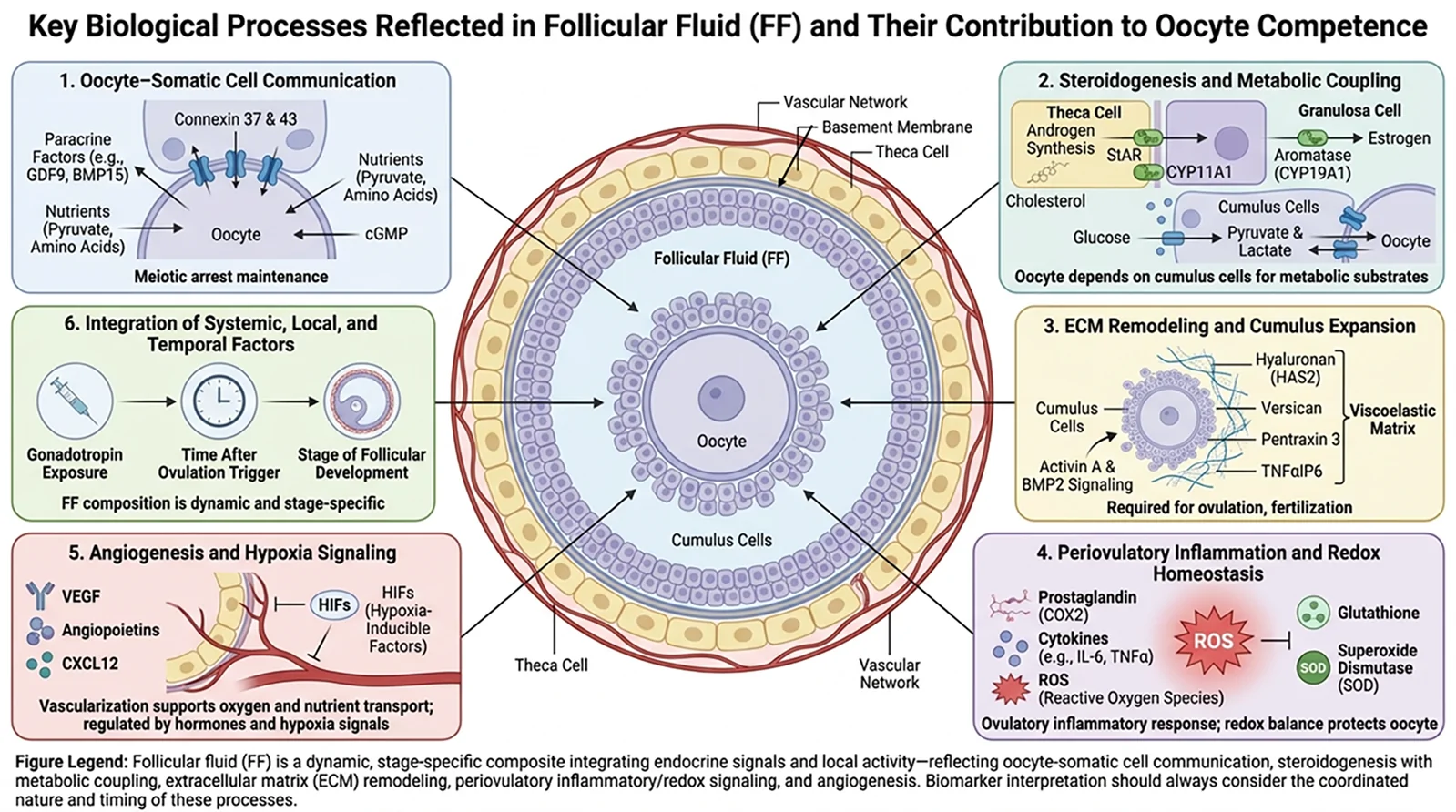
Key biological processes reflected in follicular fluid and their contributions to oocyte competence. Follicular fluid (FF) represents a dynamic, stage-dependent integration of systemic endocrine input and local activity within the oocyte–cumulus–granulosa–theca cell unit. (1) Bidirectional oocyte–somatic cell communication is mediated by connexin 37- and connexin 43-containing gap junctions, which facilitate the transfer of nutrients and cyclic guanosine monophosphate, while oocyte-derived GDF9 and BMP15 provide paracrine feedback regulating somatic-cell function and meiotic arrest. (2) Steroidogenesis follows the two-cell model, in which theca-cell-derived androgens are converted to estrogens by granulosa-cell aromatase; cumulus cells also metabolize glucose into pyruvate and lactate to support oocyte energy metabolism. (3) Cumulus expansion requires coordinated extracellular-matrix synthesis and organization involving hyaluronan/HAS2, versican, pentraxin 3, and TNFαIP6 under the regulation of factors including activin A and BMP2. (4) Periovulatory inflammation involves cytokines and COX-2-dependent prostaglandin synthesis, accompanied by reactive oxygen species generation; antioxidant systems, including glutathione and superoxide dismutase, maintain redox homeostasis and protect the oocyte from oxidative injury. (5) Follicular vascularization, regulated by VEGF, angiopoietins, CXCL12, and hypoxia-inducible factors, supports oxygen, nutrient, and hormonal delivery to the developing follicle. (6) The concentrations and biological significance of FF biomarkers are further influenced by gonadotropin exposure, the interval after ovulation triggering, and the stage of follicular development. Accordingly, FF biomarkers should be interpreted as coordinated pathway-level signatures rather than isolated analytes. BMP, bone morphogenetic protein; cGMP, cyclic guanosine monophosphate; COX-2, cyclooxygenase-2; CXCL12, C-X-C motif chemokine ligand 12; ECM, extracellular matrix; GDF9, growth differentiation factor 9; HAS2, hyaluronan synthase 2; HIF, hypoxia-inducible factor; IL-6, interleukin-6; ROS, reactive oxygen species; SOD, superoxide dismutase; StAR, steroidogenic acute regulatory protein; TNFα, tumor necrosis factor-α; TNFαIP6, tumor necrosis factor-α-induced protein 6; VEGF, vascular endothelial growth factor.

#### 2.2.2 Steroidogenesis and metabolic coupling

Follicular steroidogenesis requires coordinated activity between theca and granulosa cells. Under LH stimulation, theca cells generate androgens, which cross the follicular basement membrane and serve as substrates for FSH-induced aromatase in granulosa cells. Steroidogenic acute regulatory protein (StAR) controls the rate-limiting transfer of cholesterol into mitochondria, where cytochrome P450 cholesterol side-chain cleavage enzyme, encoded by *CYP11A1*, converts cholesterol to pregnenolone. Pregnenolone is subsequently converted to progesterone by 3β-hydroxysteroid dehydrogenase, whereas aromatase, encoded by *CYP19A1*, converts theca-derived androgens to estrogens.

During the preovulatory phase, steroidogenic differentiation must be coordinated to support estradiol synthesis while avoiding premature luteinization. In human granulosa-cell models, TGF-β1 downregulated StAR expression and reduced progesterone production through SMAD3- and ERK1/2-dependent signaling (44). Activins A, B, and AB similarly enhanced aromatase expression and estradiol production while suppressing StAR expression and progesterone synthesis. The inhibitory effect on StAR was mediated predominantly through ALK4–SMAD2–SMAD4 signaling (45). GDF8 also decreased StAR expression and progesterone production through ALK5-dependent SMAD3 and ERK1/2 signaling and enhanced gonadotropin-induced aromatase activity in cultured human granulosa-lutein cells. (46). AMH exerts a different regulatory effect. In primary human granulosa-lutein cells, it attenuated FSH-induced adenylyl cyclase activation, aromatase expression, and estradiol production through anti-Müllerian hormone receptor type 2 (47). These findings demonstrate that members of the TGF-β superfamily can differentially regulate estrogenic and progestogenic pathways according to ligand identity, receptor usage, granulosa-cell differentiation, and gonadotropin exposure (**Figure 2**).

These mechanistic data do not, however, establish that the FF concentrations of TGF-β1, activins, GDF8, or AMH collectively determine a clinically interpretable estradiol-to-progesterone ratio. The concentration of each ligand may not correspond directly to receptor occupancy or downstream signaling because bioactivity is modified by binding proteins, antagonists, receptor abundance, and post-translational processing. Moreover, FF steroid concentrations vary with follicular size, ovarian stimulation, trigger regimen, and the interval between triggering and aspiration. The proposed combination of growth factors, estradiol, progesterone, and steroidogenic intermediates is therefore biologically plausible but requires prospective validation in individually aspirated, oocyte-matched follicles.

Metabolic coupling is equally important. The oocyte has limited capacity to metabolize glucose and depends on cumulus cells to convert glucose into pyruvate and lactate. Cumulus cells also provide amino acids, nicotinamide adenine dinucleotide precursors, and other substrates through gap junctions and paracrine exchange (11). Consequently, metabolite profiles should be interpreted together with steroidogenic and somatic-cell markers rather than as independent determinants of competence.

#### 2.2.3 ECM remodeling and cumulus expansion

Periovulatory follicular remodeling requires coordinated reorganization of the follicular wall and expansion of the cumulus–oocyte complex. The expanded cumulus matrix is rich in hyaluronan synthesized predominantly by HAS2. Hyaluronan is stabilized by matrix-associated proteins, including versican, pentraxin 3, inter-α-trypsin inhibitor family members, and tumor necrosis factor α (TNFα)-induced protein 6. These components collectively generate a viscoelastic matrix required for cumulus expansion, ovulation, oviductal transport, and fertilization (3, 23, 24).

Multiple intraovarian signals converge on this matrix-remodeling program. Activin A increased HAS2 and versican expression and promoted hyaluronan production in primary and immortalized human granulosa-lutein cells through ALK4–SMAD2/3–SMAD4 signaling (7). BMP2 also increased hyaluronan production by inducing HAS2; this response involved ALK2/ALK3–SMAD signaling and was mediated partly through connective tissue growth factor. Other BMPs can activate both canonical SMAD1/5/8 and noncanonical SMAD2/3 pathways, producing ligand-specific effects on hyaluronan synthesis (3).

ECM remodeling also involves proteolytic enzymes and their inhibitors, including matrix metalloproteinases and tissue inhibitors of metalloproteinases. Nevertheless, concentrations measured in bulk FF may reflect synthesis, release from the follicular wall, substrate binding, or tissue turnover and may not directly quantify functional remodeling. A mechanistically coherent biomarker panel could therefore include hyaluronan, pentraxin 3, versican fragments, TNFα-induced protein 6, and selected protease–inhibitor pairs. Its clinical value would need to be assessed against follicle-specific outcomes rather than embryo morphology alone (**Figure 2**).

#### 2.2.4 Periovulatory inflammation, oxidative stress, and prostaglandin pathways

Ovulation resembles a localized, self-limited inflammatory response characterized by cytokine production, prostaglandin synthesis, vascular permeability, cumulus expansion, proteolysis, and follicular-wall rupture (17). Periovulatory inflammation refers to the coordinated, transient, and physiologically regulated inflammatory-like response surrounding ovulation, involving cytokines, prostaglandins, immune-cell recruitment, proteolysis, vascular changes, and controlled reactive oxygen species generation. It is distinguished from persistent or pathological inflammation. Prostaglandin is a central mediator of this program, but it should not be described solely as a trigger of follicular rupture. It contributes to cumulus expansion, oocyte maturation, follicular remodeling, and luteal differentiation. Its synthesis is driven principally by prostaglandin-endoperoxide synthase 2, also known as cyclooxygenase 2, followed by terminal prostaglandin E synthases (17).

Several pathways converge on prostaglandin-endoperoxide synthase 2 expression in human granulosa cells. Amphiregulin, betacellulin, and epiregulin increased cyclooxygenase 2 expression and prostaglandin E2 production through epidermal growth factor (EGF) receptor–ERK1/2 signaling (48). Nitric oxide and cyclic guanosine monophosphate also promoted cyclooxygenase 2 expression and prostaglandin E2 synthesis through cAMP response element-binding protein signaling (49). In addition, interleukin-6 trans-signaling through the interleukin-6/soluble interleukin-6 receptor complex activated JAK2–STAT3 signaling, increased prostaglandin-endoperoxide synthase 2 expression, and enhanced prostaglandin E2 production in primary and immortalized human granulosa-lutein cells. Suppressor of cytokine signaling 3 provided negative feedback within this pathway (50).

Reactive oxygen species also participate in ovulatory signaling, but excessive oxidative stress can impair mitochondrial function, meiotic-spindle integrity, membrane lipids, DNA, and oocyte developmental competence. FF therefore contains both pro-oxidant products and antioxidant defenses, including glutathione-related enzymes, superoxide dismutase, catalase, vitamins, and small-molecule antioxidants (16). The biological effect depends on redox balance rather than on the absolute concentration of a single oxidative marker (**Figure 2**).

Taken together, prostaglandin E2, cytokines, nitric-oxide metabolites, lipid-peroxidation products, and antioxidant-capacity measures may characterize distinct dimensions of the periovulatory inflammatory–redox state. However, their temporal sensitivity is substantial: samples collected after an ovulatory trigger capture an induced periovulatory response rather than the unstimulated follicular environment. This timing must be standardized in biomarker studies.

#### 2.2.5 Angiogenesis and hypoxia signaling

The granulosa-cell compartment is avascular, whereas the surrounding theca layer develops an extensive capillary network during antral follicle growth. Perifollicular vascularization facilitates the delivery of oxygen, gonadotropins, cholesterol, and metabolic substrates and supports the removal of cellular products. Angiogenesis and vascular permeability are regulated by vascular endothelial growth factor (VEGF), angiopoietins, fibroblast growth factors, hypoxia-inducible factors, and chemokines such as CXCL12 (28, 29). VEGF is detectable in human FF and has been associated with perifollicular blood flow in some IVF cohorts (29). Nevertheless, associations between FF VEGF and oocyte or embryo outcomes have been inconsistent. High concentrations may indicate effective vascular support, but they may also reflect relative hypoxia, increased vascular permeability, or an exaggerated response to ovarian stimulation (30). Its interpretation is therefore context-dependent and unlikely to be clinically useful as an isolated biomarker.

Evidence for angiogenin is less directly applicable to FF biomarker development. Its causal role in promoting angiogenesis and primordial-follicle survival was demonstrated in mesenchymal-stromal-cell-assisted xenotransplantation of human ovarian cortical tissue (34). This model concerns ischemic revascularization of transplanted primordial follicles rather than the physiology of individual antral follicles during IVF. It therefore supports the biological plausibility of angiogenin as an ovarian angiogenic factor but does not establish FF angiogenin as a marker of oocyte competence. The latter would require direct measurement in individually matched follicles and independent association with fertilization, blastocyst development, euploidy, implantation, or live birth.

Overall, angiogenic markers should be interpreted jointly with indicators of hypoxia, oxidative stress, perifollicular blood flow, and follicular maturity. This integrated approach may distinguish adaptive vascularization from pathological hypoxic signaling more effectively than measurement of VEGF or angiogenin alone (**Figure 2**).

## 3 Candidate follicular-fluid biomarkers

Unless otherwise specified, the candidate biomarkers discussed in Sections 3–8 are supported primarily by mechanistic experiments, exploratory omics analyses, or relatively small observational studies. The general limitations affecting their interpretation and clinical translation, including sampling heterogeneity, analytical variability, outcome inconsistency, clustering of follicles within patients, and insufficient external validation, are addressed collectively in Section 10. Individual subsections therefore emphasize only limitations specific to the biomarker or pathway being discussed.

### 3.1 Steroid hormones

Intrafollicular concentrations of estradiol, progesterone, and androgens were among the earliest biomarkers investigated as indicators of follicular maturity and oocyte competence. These hormones reflect the integrated steroidogenic activity of theca and granulosa cells in response to gonadotropins and intraovarian regulators. However, their associations with oocyte maturation, fertilization, embryo development, and pregnancy have varied substantially across studies (35, 36).

#### 3.1.1 Estradiol

In the two-cell, two-gonadotropin model, LH stimulates theca cells to produce androstenedione and testosterone, which diffuse into the granulosa-cell compartment and are converted to estradiol by FSH-induced aromatase. Intrafollicular estradiol generally increases during antral-follicle development and reflects granulosa-cell differentiation and aromatase activity. Although estradiol-rich follicles have traditionally been considered more mature, associations between FF estradiol and oocyte nuclear maturity, fertilization, embryo morphology, or pregnancy have been inconsistent (35, 36). Estradiol concentrations are affected by follicular size, granulosa-cell number, gonadotropin exposure, ovarian reserve, infertility diagnosis, and the stimulation regimen. Moreover, a high estradiol concentration may indicate active steroidogenesis without necessarily reflecting cytoplasmic maturation, chromosomal competence, or mitochondrial integrity of the enclosed oocyte. FF estradiol is therefore more appropriately interpreted as a marker of follicular estrogenic activity than as an independent measure of oocyte quality.

#### 3.1.2 Progesterone

Progesterone production increases after the LH or hCG signal initiates granulosa-cell luteinization. It is important to distinguish circulating progesterone measured before ovulation triggering from progesterone measured in FF at oocyte retrieval. Premature elevation of serum progesterone before triggering has primarily been associated with advancement of endometrial maturation and impaired embryo–endometrial synchrony in fresh-transfer cycles. Its clinical implications therefore relate predominantly to endometrial receptivity rather than to a direct detrimental effect on oocyte quality.

FF progesterone, in contrast, reflects the periovulatory steroidogenic state of an individual follicle after trigger exposure. A systematic review and meta-analysis of 13 studies encompassing 1,009 individually aspirated follicles found that FF progesterone concentrations were approximately one-third higher in follicles whose oocytes underwent normal fertilization than in those with fertilization failure (37). Nevertheless, substantial heterogeneity in stimulation regimens, progesterone assays, follicle selection, and fertilization methods precluded the establishment of a clinically applicable threshold.

The progesterone-to-estradiol ratio has been proposed as an index of follicular luteinization or maturity. However, the biological meaning of this ratio depends heavily on sampling time and trigger exposure, and its association with embryo competence or live birth has not been validated consistently. It should therefore be treated as a descriptive endocrine phenotype rather than an established “follicular health index” (51).

#### 3.1.3 Androgens

Theca cells synthesize androstenedione and testosterone from cholesterol-derived precursors under LH stimulation. These androgens act both as substrates for aromatization and as ligands for the androgen receptor in granulosa cells. Physiological androgen signaling may enhance FSH responsiveness during early follicular development, whereas excessive androgen exposure may disrupt granulosa-cell differentiation, metabolism, and oocyte–somatic-cell communication.

Altered FF androgen profiles are frequently reported in polycystic ovary syndrome (PCOS), but these differences primarily characterize the endocrine phenotype of the disorder. They have not yielded a reproducible threshold capable of predicting the developmental outcome of an individual oocyte. Interpretation is further complicated by PCOS phenotype, body mass index, insulin resistance, ovarian stimulation, and analytical differences among immunoassays and mass-spectrometry platforms (25, 26, 52).

#### 3.1.4 Methodological implications

Steroid concentrations may vary substantially among follicles from the same patient. In a prospective analysis, within-patient variability in FF estradiol was approximately twice the between-patient variability, while appreciable interpatient variation was also observed for progesterone and testosterone (53). Pooling FF from multiple follicles therefore obscures follicle-specific signals and prevents reliable linkage between a biochemical measurement and its corresponding oocyte or embryo. Conversely, analyses of individual follicles introduce clustering because several follicles originate from the same patient. This dependency must be addressed using multilevel or mixed-effects models.

No FF steroid threshold has been independently validated as a robust predictor of clinical pregnancy or live birth. Steroids may nevertheless provide valuable biological context when incorporated into panels that also measure granulosa-cell function, inflammation, redox state, metabolism, and ECM remodeling.

### 3.2 Growth factors and cytokines

FF contains growth factors and cytokines derived from the oocyte, granulosa cells, theca cells, endothelial cells, immune cells, and systemic circulation. These molecules regulate angiogenesis, steroidogenesis, cumulus expansion, inflammation, and cell survival. Their biological effects depend not only on concentration but also on receptor abundance, binding proteins, soluble receptors, antagonists, post-translational processing, and interactions with other pathways. Accordingly, immunoreactive concentration does not necessarily equal biological activity.

#### 3.2.1 Vascular endothelial growth factor

VEGF is produced by granulosa and theca cells in response to gonadotropin signaling and hypoxic stimuli. It regulates angiogenesis and vascular permeability in the perifollicular theca layer, thereby supporting the delivery of oxygen, gonadotropins, cholesterol, and metabolic substrates. FF VEGF has been associated with perifollicular blood flow in some studies, supporting its relevance to follicular vascularization (54). Its relationship with developmental competence is nevertheless inconsistent. One study found positive associations between FF VEGF, patient age, and gonadotropin consumption but no association with IVF outcome (38). Other studies reported inverse associations with embryo morphology, possibly because high VEGF reflected relative hypoxia rather than optimal vascular support (39). Conversely, cohorts linking greater perifollicular vascularity to improved fertilization and pregnancy have suggested a favorable angiogenic interpretation (54).

These apparently contradictory findings emphasize the context-dependent nature of VEGF. Its concentration may reflect adequate vascular development, a compensatory response to hypoxia, or stimulation-induced vascular permeability. VEGF should therefore be interpreted together with perifollicular blood flow, follicular size, hypoxia-related markers, and stimulation intensity rather than as an isolated biomarker.

#### 3.2.2 Insulin-like growth factors and their binding proteins

The IGF system is composed of IGF-I and IGF-II, six high-affinity IGF-binding proteins (IGFBP-1 through IGFBP-6), and the binding-protein proteases, particularly pregnancy-associated plasma protein-A (PAPP-A). Because most IGF is protein-bound, total IGF-I concentration may provide an incomplete measure of biological activity. Ratios incorporating IGF-I and its binding proteins, or direct measurements of free IGF, may be more informative. In a small IVF cohort, the IGF-I/IGFBP-1 ratio in both serum and FF was higher among women who became pregnant, whereas inhibin B was associated with the number of retrieved oocytes but not pregnancy (55). These findings were exploratory and derived from a limited patient-level analysis rather than prospective validation of follicle-specific outcomes.

Studies relating perifollicular vascularity, FF IGF-I, and embryo quality have also yielded suggestive but inconsistent results. The IGF axis is influenced by insulin resistance, nutritional status, hepatic production, follicular protease activity, and ovarian stimulation. Future panels should therefore consider IGF-I or IGF-II together with relevant binding proteins, pregnancy-associated plasma protein-A activity, and metabolic covariates rather than measuring total IGF-I alone.

#### 3.2.3 Transforming growth factor-β superfamily members

Members of the TGF-β superfamily, including activins, AMH, growth differentiation factors, BMPs and GDFs, are detectable in human FF and have established mechanistic roles in granulosa-cell differentiation, steroidogenesis, ECM formation, cumulus expansion, and oocyte–somatic-cell communication. However, most mechanistic evidence derives from primary or immortalized granulosa-cell cultures and does not by itself establish clinical biomarker performance. GDF8 has been detected in human FF and shown experimentally to reduce StAR expression and progesterone production in luteinized human granulosa cells (19). An inverse correlation between GDF8 and progesterone concentrations has also been reported in FF (20).

In women with PCOS undergoing IVF, higher FF GDF8 and lower progesterone were associated with lower pregnancy rates (21). These observations support a relationship between GDF8 and the steroidogenic phenotype of PCOS, but they do not demonstrate that GDF8 independently predicts the competence of individual oocytes or that it causes implantation failure. The reported decline in serum GDF8 between ovulation triggering and oocyte retrieval should likewise be interpreted as a cycle-level endocrine observation rather than a follicle-specific biomarker. Associations with pregnancy may be confounded by PCOS status, ovarian response, serum progesterone, embryo quality, and transfer strategy.

BMP4 and BMP7 have been shown in human granulosa-cell models to reduce pentraxin 3 expression through receptor-specific SMAD1/5/8–SMAD4 signaling (18). These results demonstrate mechanistic convergence on a cumulus-matrix-related gene, but pentraxin 3 has several cellular sources and is dynamically regulated during the periovulatory period. Suppression of pentraxin 3 in luteinized mural granulosa-cell cultures should not be equated directly with impaired cumulus expansion *in vivo*. BMP4 and BMP7 can also modulate activin-subunit expression and other granulosa-cell functions. Nevertheless, evidence for a BMP4/BMP7–activin A axis is predominantly mechanistic and does not establish that the corresponding FF concentrations form a clinically validated biomarker combination. These pathways are best presented as a biological framework for panel development rather than as established predictors of IVF success (22).

#### 3.2.4 Sources of inconsistency across studies

No single FF growth factor or cytokine has demonstrated sufficiently reproducible discrimination and calibration to support routine prediction of oocyte competence, clinical pregnancy, or live birth (4, 32, 33). This limited reproducibility reflects substantial clinical, biological, and methodological heterogeneity across studies. Patient characteristics, including age, ovarian reserve, body mass index, infertility diagnosis, PCOS phenotype, and metabolic status, may independently influence both FF composition and reproductive outcomes (4, 32, 33, 56). Treatment-related variability further arises from differences in gonadotropin type and dose, pituitary-suppression strategy, ovulation-trigger formulation, and the interval between triggering and oocyte retrieval (4, 31).

Sampling and laboratory procedures represent additional sources of variation. Studies have analyzed FF from dominant follicles, individually aspirated follicles, or samples pooled across multiple follicles, approaches that address biologically distinct questions and are not directly comparable (4, 57). Blood contamination, follicular flushing, centrifugation conditions, storage duration, and repeated freeze–thaw cycles may also alter measured analyte concentrations, emphasizing the need for standardized collection and biobanking procedures (57, 58). Furthermore, differences among immunoassays, multiplex platforms, and mass-spectrometry methods can affect analytical sensitivity, specificity, and interlaboratory comparability (4, 32, 33).

Interpretation is further complicated by inconsistent outcome definitions, which range from oocyte maturity and fertilization to embryo morphology, blastocyst development, euploidy, implantation, clinical pregnancy, and live birth. These outcomes represent different developmental stages and should not be treated as interchangeable measures of oocyte competence. Statistical analyses must also account for the hierarchical structure of ART data, because multiple follicles, oocytes, and embryos from the same patient are biologically correlated rather than independent observations (59).

### 3.3 Metabolic parameters

High-resolution mass spectrometry has been a powerful method for metabolic analyses. However, for endogenous metabolites at very low levels, such a method has been shown to be insufficient for accurate investigation in previous animal models (60). Compared to the traditional mass spectrometry (MSALL) technique, sequential window acquisition of all theoretical fragmentation spectra (SWATH™) significantly improves the hit rate of low-level endogenous metabolites, opening up the possibility to probe those metabolites that were previously impossible.

Studies have shown a unique metabolic profile in FF of PCOS patients compared to normal controls (61). Elevated free fatty acids (3-hydroxynonanoyl carnitine, eicosapentaenoic acid) were noted in the PCOS patient group. Free fatty acids have been shown to be a product of abnormal lipid metabolites that have a negative impact on cellular growth and differentiation. In another observational study, IVF patients with elevated serum progesterone concentrations (>1.5 ng/mL on the day of hCG triggering) had poorer morphological measures of oocyte quality and lower pregnancy rates than those with lower progesterone concentrations. However, this association does not establish progesterone as an independently validated predictor of oocyte competence or pregnancy outcome (62). The study also showed that lipid peroxidation products such as 4-hydroxynonenal and oxidative DNA damage markers were elevated in groups with worse oocyte quality. These findings can be interpreted as dysregulated fatty acid oxidation and a reflection of impaired membrane phospholipid metabolism. These dysregulations indicate underlying mitochondrial stress and therefore lead to evidence of worse pregnancy outcomes than their counterparts.

Metabolomic profiling provides a broader representation of the follicular microenvironment than conventional single-analyte measurements. Nuclear magnetic resonance spectroscopy, gas or liquid chromatography–mass spectrometry, and targeted or untargeted tandem mass spectrometry have identified alterations in amino acids, carbohydrates, fatty acids, acylcarnitines, phospholipids, sphingolipids, and steroid metabolites across infertility phenotypes (4, 33). Data-independent acquisition approaches, including sequential window acquisition of all theoretical fragment-ion spectra, can improve analytical coverage and reproducibility by acquiring fragmentation data across predefined mass-to-charge windows. However, they do not eliminate the major challenges of FF metabolomics. Low-abundance metabolites still require appropriate extraction, chromatographic separation, internal standards, quality-control samples, correction for batch effects, and preferably targeted quantitative validation. Putative metabolite annotations based only on accurate mass and database matching should not be treated as confirmed identities without comparison to authentic standards.

A pilot SWATH-based study involving 19 women with PCOS and 21 controls reported higher FF concentrations of 3-hydroxynonanoyl carnitine, eicosapentaenoic acid, deoxycorticosterone, phenylalanine, and leucine, together with lower lysophosphatidylcholines and phytosphingosine in PCOS (61). These findings suggest disturbances in fatty-acid oxidation, phospholipid turnover, amino-acid metabolism, and steroidogenesis. However, they primarily discriminate a PCOS phenotype from controls and should not be interpreted as evidence that any individual metabolite predicts oocyte competence. The small sample size, multiple testing, and absence of external validation further limit clinical translation. Another SWATH-based study compared women with serum progesterone above or below 1.5 ng/mL on the trigger day and reported altered FF metabolites together with increased 4-hydroxynonenal and 8-hydroxy-2′-deoxyguanosine in the higher-progesterone group (62). Although these findings are compatible with lipid peroxidation and oxidative DNA damage, the observational design cannot establish that progesterone caused oxidative stress or impaired oocyte quality. Elevated progesterone may instead identify altered follicular maturation, ovarian response, or stimulation exposure that independently affects the FF metabolome.

More broadly, lipid and acylcarnitine abnormalities may indicate altered β-oxidation, mitochondrial substrate handling, or membrane remodeling, but their biological effects are concentration- and lipid-species-dependent. For example, eicosapentaenoic acid should not be categorized generically as a harmful free fatty acid, because it is an omega-3 polyunsaturated fatty acid with context-dependent metabolic and inflammatory effects. Recent systematic reviews confirm recurrent alterations in FF lipid, amino-acid, energy-metabolism, and oxidative-stress pathways across PCOS, endometriosis, diminished ovarian reserve, and reproductive aging (33, 63). Nevertheless, results vary substantially by diagnosis, analytical platform, stimulation regimen, sample pooling, and outcome definition. Metabolomic signatures therefore remain promising discovery tools rather than validated clinical tests.

## 4 Granulosa cell–associated signaling molecules

Granulosa cells integrate endocrine signals from gonadotropins with paracrine inputs from the oocyte, theca cells, and surrounding somatic cells. Their secreted products therefore provide mechanistically informative readouts of follicular maturation.

### 4.1 EGF-like growth factors: amphiregulin, epiregulin, and betacellulin

The LH surge induces the expression of EGF-like ligands in mural granulosa cells. AREG, EREG, and BTC bind to the EGF receptor (EGFR/ERBB1) and transmit the ovulatory signal to the cumulus–oocyte complex. Among these ligands, AREG has the strongest evidence as a measurable human follicular-fluid biomarker. Its concentration increases markedly following human chorionic gonadotropin (hCG) administration, whereas EREG is detected less consistently and BTC may remain below the detection limits of some proteomic platforms (40, 64). Thus, the three ligands should not be considered analytically equivalent.

Experiments using primary and immortalized human granulosa-lutein cells have shown that AREG, EREG, and BTC increase pentraxin 3 transcription and protein accumulation through EGFR-dependent activation of ERK1/2 (65). Because pentraxin 3 contributes to the organization of the hyaluronan-rich cumulus extracellular matrix, this pathway provides a plausible connection between periovulatory EGF signaling and cumulus expansion. The same ligands also induce cyclooxygenase-2 and prostaglandin E_2_ production through the EGFR–ERK1/2 pathway (48). These findings place the EGF-like factors upstream of two complementary periovulatory processes: cumulus-matrix organization and prostaglandin-mediated ovulatory responses.

AREG is therefore a promising marker of follicular exposure to an LH-like stimulus. Nevertheless, its association with fertilization and embryo development has been inconsistent, and its concentration is strongly influenced by trigger formulation and the trigger-to-retrieval interval (40, 64). AREG, EREG, and BTC may therefore reflect periovulatory signaling activity; however, their predictive value for oocyte competence has not been independently validated (**Figure 3**).

**Figure 3:**
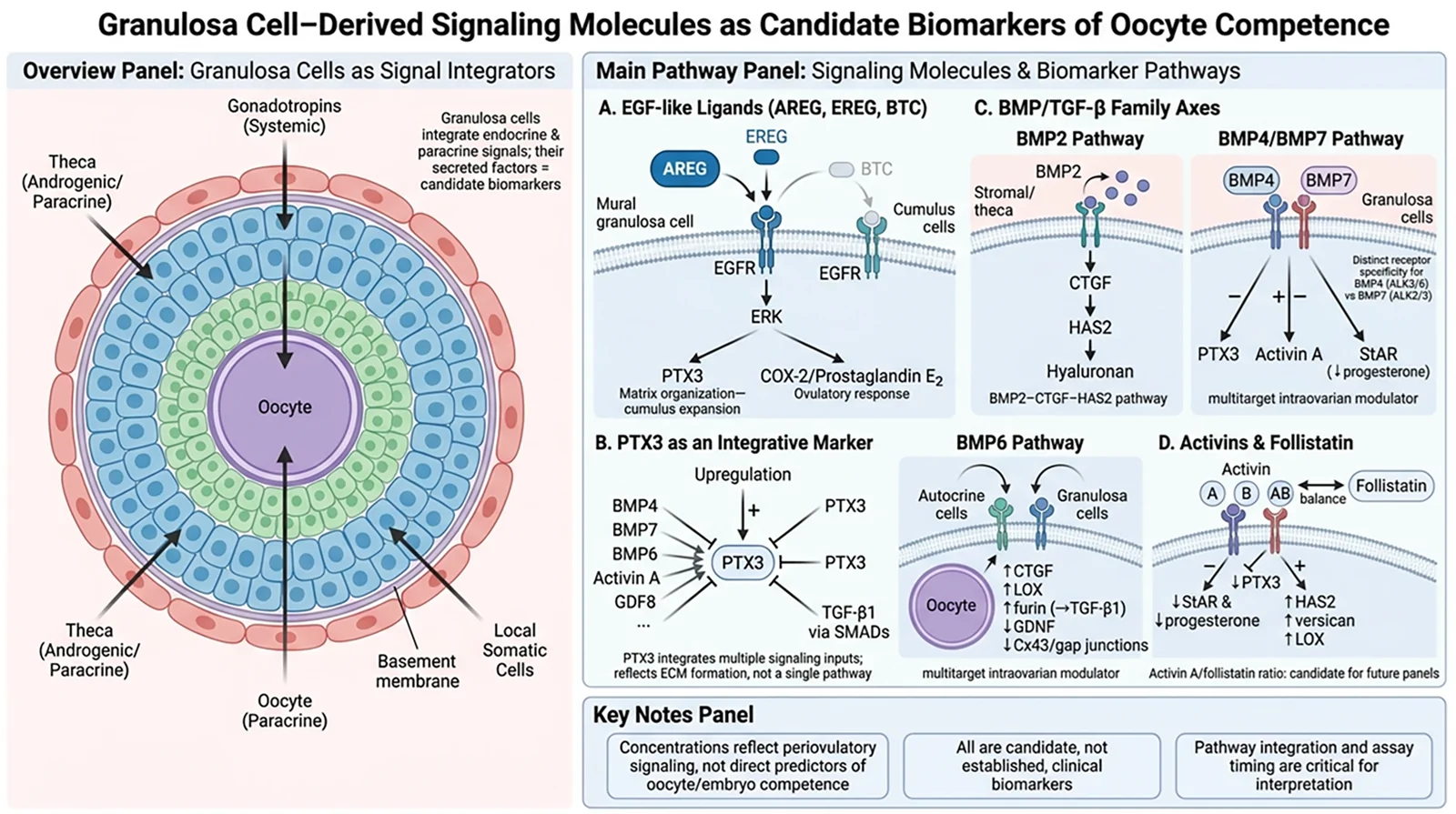
Granulosa cell-associated signaling networks as candidate biomarkers of oocyte competence. Granulosa cells integrate systemic gonadotropin stimulation with paracrine signals derived from theca cells, the oocyte, and neighboring somatic cells, generating molecular outputs that may be detected in follicular fluid or granulosa/cumulus-cell samples. **(A)** The periovulatory EGF-like ligands AREG, EREG, and BTC activate EGFR–ERK signaling in mural granulosa and cumulus cells, inducing PTX3-dependent cumulus-matrix organization and COX-2-mediated prostaglandin E_2_ production. **(B)** PTX3 represents an integrative marker of cumulus extracellular-matrix formation whose expression reflects the balance between stimulatory periovulatory signals and inhibitory inputs from BMP4, BMP6, BMP7, activin A, GDF8, and TGF-β1-associated pathways. **(C)** BMP/TGF-β family pathways regulate multiple follicular processes. BMP2 promotes CTGF, HAS2, and hyaluronan production, whereas BMP4 and BMP7 suppress PTX3 and StAR-dependent progesterone synthesis while stimulating activin A production. BMP6 regulates CTGF and LOX expression, furin-dependent TGF-β1 production, GDNF signaling, and Cx43-mediated gap-junction communication. **(D)** The net biological activity of activins A, B, and AB is modulated by follistatin. Increased activin signaling suppresses PTX3, StAR, and progesterone production while enhancing HAS2, versican, and LOX expression; consequently, the activin-to-follistatin balance may provide greater physiological information than either analyte alone. Arrows indicate activation or increased expression, whereas blunt-ended lines and minus signs indicate inhibition or decreased expression. These molecules remain candidate rather than clinically validated biomarkers: their concentrations primarily reflect pathway activity and must be interpreted according to follicular stage, sample timing, and the broader signaling context. AREG, amphiregulin; BMP, bone morphogenetic protein; BTC, betacellulin; COX-2, cyclooxygenase-2; CTGF, connective tissue growth factor; Cx43, connexin 43; ECM, extracellular matrix; EGF, epidermal growth factor; EGFR, epidermal growth factor receptor; EREG, epiregulin; ERK, extracellular signal-regulated kinase; GDF8, growth differentiation factor 8; GDNF, glial cell line-derived neurotrophic factor; HAS2, hyaluronan synthase 2; LOX, lysyl oxidase; PTX3, pentraxin 3; SMAD, suppressor of mothers against decapentaplegic; StAR, steroidogenic acute regulatory protein; TGF-β1, transforming growth factor-β1.

### 4.2 Pentraxin 3 as a mechanistically integrative candidate biomarker

Pentraxin 3 is an essential structural component of the cumulus extracellular matrix. By interacting with the heavy chains of inter-α-trypsin inhibitor, pentraxin 3 facilitates the organization and stabilization of the hyaluronan-rich matrix surrounding the oocyte. Pentraxin 3 is measurable in follicular fluid, although its concentration may reflect contributions from cumulus cells, mural granulosa cells, and inflammatory cells within the periovulatory follicle (66). Pentraxin 3 is mechanistically attractive because its expression integrates several signaling pathways. EGF-like ligands stimulate pentraxin 3 expression through EGFR–ERK1/2 signaling, whereas BMP4, BMP7, BMP6, activin A, GDF8, and TGF-β1 have been reported to suppress its expression in cultured human granulosa-lutein cells through distinct SMAD-dependent mechanisms (6, 65, 67, 68). Pentraxin 3 therefore represents a downstream convergence point rather than a readout of a single signaling pathway.

Clinical evidence, however, remains inconsistent. An early study found higher pentraxin 3 transcript abundance in cumulus cells associated with oocytes that subsequently developed into morphologically normal embryos (69). Other studies found no relationship between cumulus-cell pentraxin 3 expression and oocyte maturity or embryo quality (70). Moreover, cumulus-cell transcript abundance and follicular-fluid protein concentration are not interchangeable measurements. Pentraxin 3 should therefore be described as a mechanistically integrative candidate biomarker whose predictive discrimination and incremental clinical value remain to be established (**Figure 3**).

### 4.3 BMP/TGF-β superfamily signaling components

#### 4.3.1 BMP2: regulation of hyaluronan production through connective tissue growth factor

BMP2 is expressed within the ovarian somatic compartment and has been implicated in follicular differentiation, extracellular matrix remodeling, and luteal function. In primary and immortalized human granulosa-lutein cells, BMP2 increases connective tissue growth factor, also known as cellular communication network factor 2, and subsequently upregulates HAS2. This signaling sequence increases hyaluronan production through ALK2/ALK3-mediated, SMAD-dependent signaling (24). These findings support a BMP2–CTGF–HAS2 axis involved in cumulus-matrix formation. Nevertheless, evidence that follicular-fluid concentrations of BMP2 or CTGF independently predict oocyte competence remains limited. Claims concerning a BMP2–BAMBI interaction should also be presented separately unless directly supported by human follicular-fluid or granulosa-cell data. At present, this pathway is better regarded as a mechanistic biomarker axis than as a validated clinical panel (**Figure 3**).

#### 4.3.2 BMP4 and BMP7: paracrine regulators of granulosa-cell function

BMP4 and BMP7 are produced predominantly by ovarian stromal and theca-cell compartments and exert paracrine effects on granulosa cells. Both ligands suppress pentraxin 3 expression through SMAD1/5/8–SMAD4 signaling, although they use partially different type I receptor combinations: BMP4 signals through ALK3 and ALK6, whereas BMP7 predominantly uses ALK2 and ALK3 (18). In the same study, follicular-fluid BMP4 and PTX3 concentrations were negatively correlated, providing limited but direct support for the biological relevance of this pathway *in vivo*.

BMP4 and BMP7 also increase activin A production by upregulating the inhibin βA subunit and the proprotein convertase furin (71). This BMP4/7–furin–activin A pathway constitutes a potential theca-to-granulosa paracrine circuit. In addition, both BMPs suppress steroidogenic acute regulatory protein expression and progesterone production through ALK3–SMAD1/5/8–SMAD4 signaling (72). These observations are consistent with an anti-luteinization role.

BMP4 and BMP7 have also been reported to reduce connexin 43 expression and granulosa-cell gap-junctional communication. This finding should not be interpreted as direct evidence of impaired oocyte–cumulus communication, because connexin 43 predominantly mediates communication between granulosa or cumulus cells, whereas connexin 37 is the principal connexin at the oocyte–cumulus interface. Collectively, BMP4 and BMP7 coordinate ECM composition, activin production, steroidogenesis, and somatic-cell communication. Their clinical utility will require individual-follicle measurements linked to oocyte and embryo outcomes (**Figure 3**).

#### 4.3.3 BMP6: a multi-target intraovarian modulator

BMP6 is expressed by oocytes and granulosa cells and functions through autocrine and paracrine mechanisms. In human granulosa-lutein cell models, BMP6 increases CTGF expression through ALK2/ALK3-mediated SMAD signaling. CTGF subsequently contributes to increased lysyl oxidase expression, providing a potential mechanism through which BMP6 regulates extracellular-matrix crosslinking (73). BMP6 also suppresses glial cell line-derived neurotrophic factor expression through parallel SMAD1/5 and ERK1/2 pathways (74). Although GDNF has been implicated in oocyte maturation, these cell-culture findings do not establish that BMP6 prevents premature oocyte maturation *in vivo*. BMP6 additionally increases furin expression and thereby enhances the production of mature TGF-β1 in human granulosa-lutein cells (75). It can also suppress connexin 43 expression and gap-junctional intercellular communication through ALK2/ALK3–SMAD1/5–SMAD4 signaling (76).

These interactions identify BMP6 as an upstream regulator of several candidate readouts, including CTGF, lysyl oxidase, GDNF, TGF-β1, and connexin 43. However, the proposition that increased BMP6 directly causes defective cumulus expansion or poorer embryo development in women with polycystic ovary syndrome remains insufficiently established. Such disease-specific interpretations require matched follicle–oocyte studies and independent prospective validation (**Figure 3**).

#### 4.3.4 Activins and follistatins

Activins A, B, and AB are homo- or heterodimers composed of inhibin β subunits and are present within the human follicular compartment. In cultured human granulosa-lutein cells, these activins suppress steroidogenic acute regulatory protein expression and progesterone production, supporting a role in limiting premature luteinization (77). Activin A also suppresses pentraxin 3 expression through ALK4–SMAD2/3–SMAD4 signaling (6). The effects of activin A on the ECM are component specific. Despite suppressing pentraxin 3 in granulosa-cell models, activin A increases HAS2 expression, hyaluronan production, and versican accumulation through ALK4-dependent SMAD signaling. It also stimulates CTGF-dependent lysyl oxidase expression and enzymatic activity (78). These observations demonstrate that a reduction in one matrix component should not automatically be interpreted as global inhibition of cumulus expansion. The biological consequences are likely to depend on follicular stage, cell type, ligand concentration, and the balance among matrix constituents.

Follistatin binds activins with high affinity and limits their access to cell-surface receptors (79). Consequently, the molar balance between bioactive activins and follistatin may be more informative than the concentration of either component alone. Nevertheless, an activin A-to-follistatin ratio has not yet been analytically standardized or prospectively validated as a predictor of oocyte competence, pregnancy, or live birth. It should therefore be presented as a biologically plausible candidate for multianalyte evaluation rather than an established biomarker (**Figure 3**).

## 5 Oxidative stress and mitochondrial function biomarkers

### 5.1 Redox balance in the follicular niche

Reactive oxygen species (ROS), including superoxide and hydrogen peroxide, are generated predominantly through mitochondrial oxidative phosphorylation and other cellular enzymatic reactions. At controlled concentrations, ROS function as signaling mediators involved in granulosa-cell steroidogenesis, meiotic resumption, cumulus expansion, and ovulation. The periovulatory follicle therefore requires a regulated degree of oxidative activity rather than complete elimination of ROS. Oxidative stress arises when ROS production exceeds the buffering capacity of enzymatic and non-enzymatic antioxidant systems, resulting in lipid peroxidation, protein oxidation, mitochondrial dysfunction, and oxidative DNA damage (16, 80).

The antioxidant network within FF includes superoxide dismutase, catalase, glutathione peroxidase, reduced glutathione, vitamins C and E, melatonin, and other low-molecular-weight scavengers. Consequently, redox status cannot be adequately represented by a single measurement. Total antioxidant capacity provides a composite estimate of antioxidant activity, whereas glutathione, malondialdehyde, protein carbonyls, 8-hydroxy-2′-deoxyguanosine (8-OHdG), and oxidized lipid products reflect distinct components of the oxidative-stress response. In women undergoing IVF, lower FF glutathione and higher 8-OHdG concentrations have been associated with reduced fertilization and good-quality blastocyst rates, although associations with clinical pregnancy have been less consistent (81).

Nitric oxide (NO) illustrates the concentration-dependent effects of reactive species in the follicle. In human granulosa-cell models, NO activates cyclic guanosine monophosphate and cAMP response element-binding protein signaling, thereby inducing cyclooxygenase-2 expression and prostaglandin E_2_ production (49). This pathway may contribute to physiological ovulatory signaling. However, when NO is produced in excess in the presence of superoxide, these radicals react to form peroxynitrite, a highly reactive nitrogen species capable of inducing lipid, protein, and DNA damage. Measurement of NO metabolites or nitrotyrosine alone therefore cannot distinguish physiological signaling from nitrosative injury without accompanying measurements of antioxidant capacity and oxidative damage.

Metabolomic studies provide additional but predominantly indirect evidence of mitochondrial and redox dysfunction. A SWATH mass-spectrometry study identified increased free fatty acids, 3-hydroxynonanoyl carnitine, and eicosapentaenoic acid, together with decreased lysophosphatidylcholine species, in FF from women with PCOS (61). Accumulation of an acylcarnitine may be consistent with incomplete fatty-acid oxidation, but it does not directly demonstrate increased mitochondrial ROS production. Similarly, reduced lysophosphatidylcholines may reflect altered phospholipase activity, membrane remodeling, cellular uptake, or oxidative degradation. These metabolites should therefore be interpreted as components of a perturbed lipid-metabolic profile rather than specific oxidative-stress markers. Notably, lysophosphatidylcholine concentrations also vary with follicular development, further emphasizing the need for adjustment for follicle size and maturation stage (74).

### 5.2 Mitochondrial quality control

Mitochondrial quality control encompasses mitochondrial biogenesis, fusion, and fission, selective removal of damaged mitochondria through mitophagy, maintenance of mitochondrial DNA (mtDNA), and regulation of respiratory-chain activity (75). These processes are essential in both the oocyte and its surrounding somatic cells. Granulosa and cumulus cells supply the oocyte with pyruvate, lactate, amino acids, and reducing equivalents, while the oocyte depends on its mitochondrial pool to generate the ATP required for meiotic maturation, fertilization, and early embryonic development (82). Potential indicators of mitochondrial function include ATP concentration, mitochondrial membrane potential, oxygen-consumption rate, mtDNA copy number, mtDNA integrity, and expression of proteins involved in mitochondrial biogenesis, dynamics, and mitophagy (77). Relevant intracellular proteins include mitochondrial transcription factor A and peroxisome proliferator-activated receptor-γ coactivator 1α for mitochondrial biogenesis; mitofusins 1 and 2 and optic atrophy 1 for fusion; dynamin-related protein 1 for fission; and PINK1, Parkin, BNIP3, and LC3-associated pathways for mitophagy (83). However, most of these measurements require oocytes or granulosa/cumulus cells and cannot be reliably inferred from unprocessed FF.

The mtDNA content of cumulus granulosa cells has been proposed as an indirect marker of the metabolic support provided to the corresponding oocyte. In an individual-oocyte study, higher cumulus granulosa-cell mtDNA copy numbers were associated with better embryo morphology but not with oocyte maturity or fertilization (84). Importantly, mtDNA content was strongly influenced by patient-level factors, including body mass index and smoking, and no universal predictive threshold could be established.

Cell-free DNA in FF may provide another indirect measure of follicular-cell stress, apoptosis, or tissue turnover. Higher FF cell-free DNA concentrations have been associated with smaller follicles, greater embryo fragmentation, and poorer embryo morphology (57). Cell-free mitochondrial DNA is particularly attractive because it may reflect mitochondrial injury and the release of mitochondrial components. A recent exploratory sequencing study identified FF cell-free mtDNA variants associated with stimulation duration, oocyte yield, and embryo quality (85). However, the small sample size, absence of independent validation, and potential confounding by leukocytes, granulosa-cell disruption, and blood contamination preclude clinical application.

Accordingly, mitochondrial quality-control biomarkers should preferably be evaluated as a coordinated panel. Such a panel might combine a measure of mitochondrial injury, such as cell-free mtDNA or 8-OHdG; a metabolic readout, such as acylcarnitines or the lactate-to-pyruvate ratio; and an antioxidant measure, such as glutathione or total antioxidant capacity. Whether this approach provides predictive information beyond age, ovarian reserve, follicle size, and embryo morphology requires prospective evaluation.

### 5.3 Clinical relevance: association with embryo development and blastocyst formation

Human studies generally support an association between an imbalanced follicular redox environment and impaired developmental competence, but the direction and magnitude of individual associations have varied. Higher FF 8-OHdG concentrations and lower glutathione levels have been associated with reduced fertilization and good-quality blastocyst formation (81). Higher malondialdehyde concentrations have also been linked to poorer cleavage or embryo-development measures in some cohorts (86). Conversely, moderate ROS or lipid-peroxidation activity has occasionally been associated with fertilization or embryo viability, consistent with the physiological requirement for controlled redox signaling (80, 87).

Composite antioxidant measurements have produced similarly heterogeneous results. Some studies found higher total antioxidant capacity in follicles yielding fertilized oocytes, whereas others reported no association or apparently lower antioxidant capacity in follicles producing embryos that survived to transfer (80, 81). These differences may reflect the biphasic biological effects of ROS, differences in antioxidant assays, timing of collection, stimulation protocols, follicle pooling, and failure to account for multiple follicles from the same woman.

Evidence for predicting blastocyst formation is more limited than evidence for fertilization or cleavage-stage morphology. Most studies were conducted before routine extended blastocyst culture, used small samples, evaluated pooled FF, or did not maintain a one-to-one link between each follicle and its resulting embryo. Furthermore, embryo morphology does not necessarily indicate chromosomal competence, implantation potential, or live birth. Current evidence therefore does not support the use of any individual oxidative-stress or mitochondrial marker for routine oocyte or embryo selection.

A more clinically informative strategy would combine markers representing complementary biological domains. For example, an integrated panel could incorporate oxidative DNA damage (8-OHdG), lipid peroxidation (malondialdehyde or F_2_-isoprostanes), antioxidant buffering (glutathione and total antioxidant capacity), mitochondrial dysfunction (acylcarnitines or cell-free mtDNA), and cellular injury (cell-free nuclear DNA).

## 6 Immune and inflammatory signatures

Ovulation exhibits several features of a controlled inflammatory response, including cytokine production, prostaglandin synthesis, vascular remodeling, ECM degradation, and recruitment or activation of immune cells (88, 89). FF inflammatory mediators may therefore provide information about periovulatory follicular activity. However, physiological ovulatory inflammation must be distinguished from chronic or excessive inflammation associated with conditions such as PCOS, endometriosis, obesity, and metabolic dysfunction.

### 6.1 Periovulatory inflammatory mediators

Multiple pathways converge on prostaglandin-endoperoxide synthase 2 (PTGS2), which encodes cyclooxygenase-2, and subsequent PGE_2_ production in human granulosa cells. Following the LH surge or ovulatory hCG administration, the EGF-like ligands AREG, EREG, and BTC activate EGFR–ERK1/2 signaling, thereby inducing PTGS2 expression and PGE_2_ synthesis (48). Among these ligands, AREG has the strongest evidence as a measurable human FF mediator, whereas EREG is detected less consistently and BTC may remain below the detection limits of some assays.

NO provides a second route to PGE_2_ production. In an immortalized human granulosa-lutein cell model, an NO donor and a cGMP analog induced PTGS2 expression and PGE_2_ production through CREB activation (49). Controlled NO production may contribute to ovulation and vascular regulation, whereas excessive NO can react with superoxide to generate peroxynitrite and cause nitrosative injury. FF NO metabolites should therefore be interpreted together with oxidative- and nitrosative-damage markers.

A third pathway involves interleukin-6 (IL-6) trans-signaling. In primary and immortalized human granulosa-lutein cells, combined IL-6 and soluble IL-6 receptor-α activated JAK2–STAT3 signaling, increased PTGS2 expression and PGE_2_ production, and induced SOCS3 as a negative-feedback regulator (50). These findings suggest that IL-6 alone may not adequately represent pathway activity. A panel incorporating IL-6, soluble IL-6 receptor-α, soluble gp130, and PGE_2_ may provide a more complete assessment, although neither an optimal IL-6-to-receptor ratio nor clinically useful thresholds have been established.

Clinical associations between FF IL-6 and IVF outcomes remain inconsistent. Lower concentrations have been associated with a greater likelihood of clinical pregnancy in some cohorts, whereas other studies have linked higher IL-6 concentrations to reduced embryo fragmentation and improved pregnancy rates (90, 91). This heterogeneity probably reflects differences in patient characteristics, stimulation protocols, sample pooling, collection timing, and assay platforms, as well as the dual physiological and pathological roles of follicular inflammation.

### 6.2 Immune–metabolic crosstalk

Immune and metabolic signaling are closely interconnected within the follicle. Cytokines can alter granulosa-cell glucose and lipid metabolism, whereas fatty acids, phospholipid derivatives, oxidative metabolites, and steroid hormones can modulate inflammatory pathways. This interaction may be especially relevant in PCOS, in which hyperandrogenism, insulin resistance, systemic low-grade inflammation, and ovarian dysfunction frequently coexist.

SWATH mass spectrometry identified increased free fatty acids, 3-hydroxynonanoyl carnitine, eicosapentaenoic acid, deoxycorticosterone, phenylalanine, and leucine, together with reduced lysophosphatidylcholines and phytosphingosine, in FF from women with PCOS (61). These alterations demonstrate metabolic dysregulation but do not necessarily indicate a uniformly proinflammatory state. Eicosapentaenoic acid may generate relatively less inflammatory lipid mediators, while lysophosphatidylcholines exert context-dependent immunomodulatory effects. These metabolites should therefore be interpreted as components of an altered follicular metabolic network rather than isolated inflammatory biomarkers.

BMP6 may represent another interface between metabolic, extracellular-matrix, and inflammatory signaling. Increased BMP6 expression has been reported in granulosa cells and FF from women with PCOS, accompanied by reduced pentraxin 3 expression (92, 93). In granulosa-cell models, BMP6 suppresses pentraxin 3 through ALK2/ALK3–SMAD1/5–SMAD4 signaling, increases furin-dependent TGF-β1 production, and stimulates CTGF expression (93, 94). These findings support interactions among BMP6, TGF-β1, pentraxin 3, and ECM remodeling. However, they do not establish a single linear proinflammatory or anti-ovulatory pathway *in vivo* because TGF-β1 and CTGF have stage- and context-dependent biological effects.

### 6.3 Inflammation-adjusted mechanism-informed candidate panels

Pentraxin 3 is both an innate immune-related protein and an essential component of the cumulus ECM. Its expression integrates several intraovarian pathways: EGF-like ligands stimulate pentraxin 3 through EGFR–ERK1/2 signaling, whereas BMP4, BMP7, BMP6, activin A, GDF8, and TGF-β1 suppress its expression through distinct SMAD-dependent pathways (6, 65, 68, 93, 49). Pentraxin 3 should therefore be considered a mechanistically integrative candidate biomarker rather than a specific marker of inflammation.

A mechanism-informed panel could combine upstream mediators such as AREG, IL-6, soluble IL-6 receptor-α, and TGF-β1 with downstream functional readouts such as PGE_2_ and pentraxin 3. Oxidative-stress markers and selected lipid metabolites could provide additional biological context. Such integration may distinguish an appropriate periovulatory inflammatory response from chronic or dysregulated inflammation more effectively than any single analyte. Nevertheless, no validated interpretative thresholds currently exist, and available evidence does not support using these biomarker patterns to select anti-inflammatory treatment or EGF-like factor supplementation.

## 7 Omics-based biomarkers

Omics technologies enable simultaneous characterization of proteins, metabolites, lipids, and RNA species within the follicular microenvironment. Unlike single-analyte measurements, these platforms can identify coordinated pathway alterations relevant to follicular maturation and oocyte competence. However, most reported signatures remain exploratory because of small cohorts, heterogeneous sampling, batch effects, high dimensionality, and limited external validation.

### 7.1 Proteomics of follicular fluid: SWATH-MS

The Sequential Windowed Acquisition of all Theoretical fragment ions mass spectrometry (SWATH-MS) is a powerful method for detailed protein analysis. Compared to traditional MS, SWATH-MS captures all fragment ions within predefined mass windows. Therefore, it has higher protein coverage and better quantification ability. SWATH-MS also offers higher sensitivity, especially in complex samples, making it a desirable method for follicular fluid analysis. Reproducibility was also highly consistent, sometimes making repeated experiments unnecessary. Due to these benefits, SWATH-MS measures proteins that reflect the functional state of the follicular environment, providing insights into ovarian physiology and factors that influence assisted reproductive technology outcomes.

Sequential window acquisition of all theoretical mass spectra mass spectrometry (SWATH-MS) is a data-independent acquisition method in which precursor ions across a defined mass-to-charge range are sequentially isolated and fragmented. The resulting fragment-ion maps allow consistent identification and label-free quantification of numerous peptides and proteins across samples (95, 96). Compared with conventional data-dependent acquisition, SWATH-MS can reduce missing measurements and improve quantitative reproducibility. Its performance nevertheless depends on sample preparation, instrument configuration, spectral-library quality, and data-processing methods. SWATH-MS is potentially useful for FF, which contains proteins derived from plasma, follicular cells, the oocyte, and immune cells. Proteomic studies have identified FF proteins involved in complement activation, coagulation, metabolism, antioxidant defense, extracellular-matrix remodeling, and growth-factor signaling (97, 98). Importantly, analysis of multiple follicles from the same woman demonstrated substantial interfollicular variation, indicating that pooled FF may obscure signals linked to individual oocytes (99).

High analytical reproducibility does not eliminate the need for technical quality control and biological replication. Randomized acquisition order, internal standards, pooled quality-control samples, batch correction, and independent validation remain essential. Discovery-phase candidates should subsequently be quantified using targeted mass spectrometry or validated immunoassays before clinical application.

### 7.2 Metabolomics and lipidomics

Metabolomics and lipidomics provide downstream functional readouts of glycolysis, the tricarboxylic acid cycle, fatty-acid oxidation, amino-acid metabolism, phospholipid remodeling, steroidogenesis, and oxidative stress. These profiles may reflect follicular physiology more directly than transcript abundance, but they are highly sensitive to diet, medication exposure, systemic metabolic status, sample handling, and storage.

In women with PCOS, SWATH-based metabolomic analysis identified increased free fatty acids, 3-hydroxynonanoyl carnitine, eicosapentaenoic acid, deoxycorticosterone, phenylalanine, and leucine, together with reduced lysophosphatidylcholines and phytosphingosine (61). These findings suggest alterations in lipid, amino-acid, and steroid metabolism but do not establish that the metabolites are specific consequences of insulin resistance or independent predictors of oocyte competence.

Lysophosphatidylcholines are particularly relevant because they participate in membrane turnover, lipid transport, inflammatory signaling, and follicular development. Their concentrations also vary with follicle size and maturation stage, limiting interpretation without appropriate adjustment (74). Metabolomic studies have additionally identified oxidative-stress- and lipid-peroxidation-related changes in women with elevated progesterone, although these findings require confirmation using targeted quantitative assays (62).

### 7.3 Transcriptomic and RNA modification signals

Transcriptomic analysis of cumulus and granulosa cells can identify the cellular regulatory programs underlying FF protein and metabolite profiles. MicroRNAs are especially attractive because they regulate gene expression post-transcriptionally and can be measured in cumulus or granulosa-cell samples obtained during oocyte retrieval. In a human *in-vitro* maturation study, glial cell line-derived neurotrophic factor (GDNF) increased the proportion of oocytes reaching metaphase II and reduced miR-145-5p expression in cultured cumulus cells (27). Cumulus-cell miR-145-5p expression was inversely associated with oocyte nuclear maturity, while mechanistic experiments showed that miR-145-5p regulated GDNF family receptor-α1, RET, EGFR, and cumulus-cell apoptosis. However, GDNF supplementation did not significantly improve fertilization, cleavage, or blastocyst formation. Thus, miR-145-5p remains a mechanistic candidate marker of cumulus-cell function and nuclear maturation rather than a validated predictor of developmental competence. The GDNF-miR-145-5p axis should be regarded as an exploratory, cell-associated research signal supported by a single human *in-vitro* maturation study. It is not currently a core follicular-fluid biomarker component and requires independent replication before inclusion in a predictive panel.

A second regulatory axis involves TGF-β1 and miR-29a. In primary and immortalized human granulosa-lutein cells, TGF-β1 suppressed miR-29a and increased lysyl oxidase expression and activity (100). Manipulation of miR-29a confirmed that its downregulation contributed to the TGF-β1-induced increase in lysyl oxidase, supporting a role in periovulatory ECM remodeling. Nevertheless, these *in vitro* findings do not establish that miR-29a predicts IVF outcomes or that its reduction represents pathological follicular fibrosis.

Both microRNAs require standardized cell isolation, RNA extraction, normalization, and RT-qPCR procedures. Neither currently has a clinically validated threshold for predicting blastocyst formation, implantation, or live birth. Because the evidence in this section concerns gene expression and microRNA-mediated regulation rather than direct chemical modification of RNA, “transcriptomic and post-transcriptional signals” is more accurate than “RNA modification signals.”

### 7.4 Multi-omics integration

Each omics layer captures a different level of follicular biology. Transcriptomics identifies regulatory programs, proteomics measures translated and secreted effectors, and metabolomics or lipidomics reflects downstream biochemical activity. Integrating these layers may therefore reveal coordinated pathway signatures that are more biologically informative and potentially more reproducible than isolated molecules (101, 102). A preliminary human study combining FF metabolomics with matched cumulus-cell transcriptomics identified concordant alterations in the tricarboxylic acid cycle, oxidative phosphorylation, cAMP and mTOR signaling, and steroid-hormone biosynthesis in women with PCOS (103). Several metabolites differentiated PCOS from control samples or correlated with fertilization and embryo-quality outcomes. However, the study was exploratory, primarily addressed PCOS classification, and lacked independent external validation.

Model development must be separated rigorously from validation. Preprocessing, normalization, imputation, feature selection, and parameter optimization should be performed exclusively within the training data to prevent information leakage and overoptimistic performance estimates (104, 105). Analyses should also account for multiple follicles nested within women and adjust for age, ovarian reserve, body mass index, infertility diagnosis, stimulation protocol, follicle size, trigger formulation, and trigger-to-retrieval interval. Omics-derived panels should progress sequentially through analytical verification, targeted-assay development, internal validation, independent external validation, and prospective clinical evaluation. Performance should be assessed using discrimination, calibration, clinical utility, and improvement over established clinical and embryological predictors (106, 107). The objective is not to construct the largest possible molecular signature, but to identify the smallest analytically robust panel that adds reproducible and clinically meaningful information.

## 8 Proposed research frameworks linked to specific IVF outcomes

The biological determinants of oocyte maturation, fertilization, embryo development, and blastocyst formation overlap but are not identical (**Table 2**). Proposed research frameworks should therefore be developed for clearly defined outcomes rather than assumed to predict the entire IVF trajectory. Importantly, most proposed FF panels are supported by mechanistic or observational evidence rather than prospective validation. Because FF is collected at oocyte retrieval, its near-term clinical application would primarily involve research, prognostic assessment, or prioritization of sibling oocytes and embryos rather than modification of the ongoing stimulation cycle. Valid studies require individual-follicle aspiration and an unambiguous link between each FF sample, its corresponding oocyte, and the resulting embryo.

FF biomarker studies should prespecify one primary outcome and report its assessment time and denominator. Oocyte maturity should be defined as metaphase II status, indicated by extrusion of the first polar body at the time of insemination or intracytoplasmic sperm injection, and reported per retrieved oocyte. Normal fertilization should be defined by the presence of two pronuclei and, when assessed, two polar bodies at the laboratory-specified fertilization check and reported per inseminated or injected mature oocyte. Cleavage-stage development should be assessed on a prespecified day using a stated morphological grading system. Blastocyst formation should be defined by development to the blastocyst stage by a specified day, generally day 5, 6, or 7, with the denominator and inclusion of delayed blastulation clearly stated. A usable or high-quality blastocyst should meet prospectively defined criteria for transfer, cryopreservation, or biopsy and should not be considered interchangeable with blastocyst formation or morphology alone. Euploidy should be defined according to the preimplantation genetic testing platform, reporting thresholds, management of mosaic results, and stated denominator. Implantation should be reported as the number of gestational sacs per embryo transferred, with fetal cardiac activity specified separately. Clinical pregnancy should be defined by ultrasonographic visualization of at least one intrauterine gestational sac, with the requirement for fetal cardiac activity explicitly stated. Ongoing pregnancy and live birth should be reported separately using a prespecified gestational-age threshold and an appropriate denominator, such as embryo transfer, oocyte retrieval, or initiated cycle. These outcomes represent distinct biological and clinical stages and should not be combined under nonspecific terms such as “oocyte quality” or “IVF success”.

### 8.1 Oocyte maturity and normal fertilization

A candidate maturation panel should capture periovulatory signaling, cumulus-matrix organization, luteinization, and neurotrophic support. AREG is the most consistently detectable EGF-like ligand in human FF and reflects LH/hCG-induced EGFR–ERK1/2 signaling. In human granulosa cells, AREG, EREG, and BTC stimulate pentraxin 3 and PTGS2-derived PGE_2_, thereby linking the ovulatory stimulus to cumulus expansion and prostaglandin production (48, 65). Because EREG is detected inconsistently and BTC may fall below assay detection limits, AREG is currently the strongest candidate among these ligands.

Pentraxin 3 is an essential component of the cumulus extracellular matrix and integrates stimulatory EGF-like signaling with inhibitory inputs from BMP4, BMP7, BMP6, activin A, GDF8, and TGF-β1. BMP4 and GDF8 may therefore provide context for interpreting pentraxin 3. GDF8 additionally suppresses StAR and progesterone production, linking cumulus-matrix regulation with luteinization status (21, 67).

The GDNF–miR-145-5p axis provides a complementary cumulus-cell marker. In human *in-vitro* maturation experiments, GDNF increased metaphase II maturation while reducing miR-145-5p expression and cumulus-cell apoptosis (27). However, the GDNF-miR-145-5p axis should be regarded as an exploratory, cell-associated research signal supported by a single human *in-vitro* maturation study. It is not currently a core FF biomarker component and requires independent replication before inclusion in a predictive panel. A parsimonious maturation panel could therefore combine AREG, pentraxin 3, PGE_2_, and either BMP4 or GDF8, and cumulus-cell miR-145-5p evaluated as an additional cell-associated module. Prediction of two-pronuclear fertilization must also incorporate sperm quality and insemination methods (**Figure 4**).

**Figure 4:**
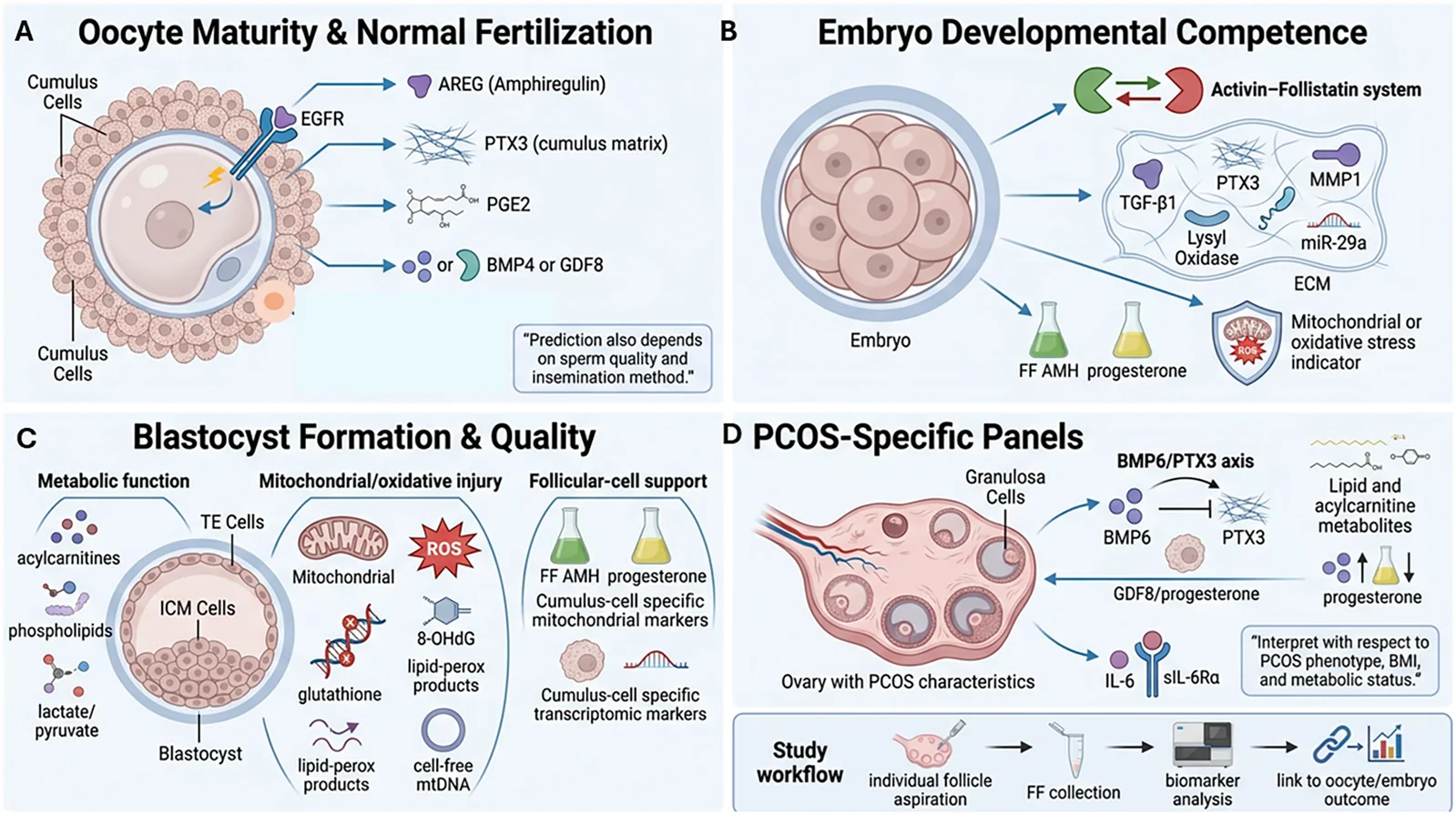
Mechanism-informed research frameworks for evaluating follicular-fluid biomarkers in relation to specific IVF outcomes. Candidate measurements are organized according to the reproductive outcome and biological pathway they are hypothesized to reflect. The illustrated combinations are not validated biomarker panels; most proposed relationships are derived from experimental studies in human granulosa-lutein or cumulus cells, whereas direct evidence from prospectively matched follicle–oocyte–embryo datasets remains limited. **(A)** Oocyte maturity and normal fertilization may be evaluated using a periovulatory signaling panel comprising AREG–EGFR activity, PTX3-mediated cumulus-matrix organization, PGE_2_ production, BMP4 or GDF8 signaling, and the GDNF–miR-145-5p axis. Fertilization prediction must additionally account for sperm quality and the insemination method. **(B)** Embryo developmental competence may be reflected by the activin–follistatin balance; an extracellular-matrix remodeling signature incorporating TGF-β1, PTX3, MMP1, lysyl oxidase, and miR-29a; FF concentrations of AMH and progesterone; and indicators of mitochondrial dysfunction or oxidative stress. **(C)** Blastocyst formation and quality may be associated with metabolic substrates and intermediates, including acylcarnitines, phospholipids, and the lactate-to-pyruvate balance; markers of mitochondrial or oxidative injury, including reactive oxygen species, glutathione, 8-hydroxy-2′-deoxyguanosine, lipid-peroxidation products, and cell-free mitochondrial DNA; and follicular-cell support indicators, including FF AMH, progesterone, and cumulus-cell-specific mitochondrial and transcriptomic markers. **(D)** Phenotype-specific assessment in PCOS may incorporate the BMP6–PTX3 and GDF8–progesterone axes, lipid and acylcarnitine metabolites, and IL-6 trans-signaling components, including soluble IL-6 receptor-α. These measurements should be interpreted according to PCOS phenotype, body mass index, and metabolic status. The proposed future study workflow requires individually aspirated follicular-fluid samples linked one-to-one with the corresponding oocyte, embryo development, and clinically relevant reproductive outcome. It should incorporate analytical verification, multilevel modeling, assessment of incremental predictive value, and independent external validation. Arrows indicate reported positive associations or pathway activation, whereas blunt-ended lines indicate experimentally observed inhibition. None of the illustrated combinations is currently suitable for clinical decision-making or embryo selection. 8-OHdG, 8-hydroxy-2′-deoxyguanosine; AMH, anti-Müllerian hormone; AREG, amphiregulin; BMP, bone morphogenetic protein; BMI, body mass index; ECM, extracellular matrix; EGFR, epidermal growth factor receptor; FF, follicular fluid; GDF8, growth differentiation factor 8; GDNF, glial cell line-derived neurotrophic factor; ICM, inner cell mass; IL-6, interleukin-6; miR, microRNA; MMP1, matrix metalloproteinase 1; mtDNA, mitochondrial DNA; PCOS, polycystic ovary syndrome; PGE_2_, prostaglandin E_2_; PTX3, pentraxin 3; ROS, reactive oxygen species; sIL-6Rα, soluble interleukin-6 receptor-α; TE, trophectoderm; TGF-β1, transforming growth factor-β1.

### 8.2 Embryo developmental competence

Embryo development depends on cytoplasmic maturation, mitochondrial function, maternal molecular reserves, and metabolic support accumulated during folliculogenesis. Candidate panels should consequently integrate endocrine, extracellular-matrix, and metabolic markers.

The activin–follistatin system is mechanistically relevant because follistatin regulates the availability of receptor-active activin. Activins suppress pentraxin 3, StAR protein, and progesterone while stimulating HAS2, versican, CTGF, and lysyl oxidase (108). Nevertheless, no clinically validated activin-to-follistatin ratio predicts embryo development, and concentrations vary with follicle size and PCOS status (109).

A related ECM module could include TGF-β1, pentraxin 3, MMP1, lysyl oxidase, and miR-29a. TGF-β1 suppresses pentraxin 3 through SNAIL, downregulates MMP1 through ID3, and increases lysyl oxidase through miR-29a suppression (68, 100, 110). These pathways characterize the balance between matrix organization, degradation, and stabilization but have not been validated as an embryo-selection panel.

Among endocrine markers, FF AMH has more extensively evaluated observational evidence than many other candidate growth factors. Individual-follicle studies have associated FF AMH with normal fertilization, embryo morphology, blastocyst development, and live birth after fresh single-embryo transfer (111, 112). Combined FF AMH and progesterone concentrations have also been associated with blastocyst development (113). Although AMH expression declines during follicular selection and is low in mural granulosa cells of large preovulatory follicles, measurable follicular-fluid AMH may integrate earlier granulosa-cell production, differences between mural and cumulus-cell compartments, and variation in follicular maturation or atresia. Individual-follicle studies have evaluated its associations with fertilization and embryo development more extensively than many other candidate growth factors; however, proposed thresholds remain inconsistent and have not demonstrated sufficient external validity or incremental clinical utility for routine application (**Figure 4**).

### 8.3 Blastocyst formation and quality

Blastocyst formation requires sustained mitochondrial ATP production, embryonic genome activation, cellular proliferation, and trophectoderm and inner-cell-mass differentiation. Although FF metabolomics may capture aspects of oocyte metabolic competence, no metabolomic panel has been independently validated for blastocyst prediction. The combination of lysophosphatidylcholines, 3-hydroxynonanoyl carnitine, eicosapentaenoic acid, and deoxycorticosterone was identified as a PCOS-associated metabolic signature rather than a blastocyst-prediction panel (61). These metabolites may reflect altered fatty-acid oxidation or phospholipid remodeling, but they do not directly demonstrate mitochondrial dysfunction.

More relevant candidate measures include FF glutathione, 8-hydroxy-2′-deoxyguanosine, lipid-peroxidation products, cell-free DNA, and cumulus-cell mitochondrial parameters. Higher 8-hydroxy-2′-deoxyguanosine and lower glutathione have been associated with reduced fertilization and good-quality blastocyst formation (81). Lower FF cell-free DNA has been associated with better embryo morphology, whereas higher cumulus-cell mitochondrial DNA content has been associated with embryo quality in some, but not all, studies (57, 84).

A candidate blastocyst panel could therefore cover three domains: metabolic function, represented by acylcarnitines, phospholipids, and lactate-to-pyruvate balance; mitochondrial or oxidative injury, represented by glutathione, 8-hydroxy-2′-deoxyguanosine, lipid-peroxidation products, and cell-free mitochondrial DNA; and follicular-cell support, represented by FF AMH, progesterone, and cumulus-cell mitochondrial or transcriptomic markers. Such panels should first be developed against usable blastocyst formation and subsequently tested against euploidy, implantation, and live birth (**Figure 4**).

### 8.4 PCOS-specific panels

PCOS is heterogeneous, and biomarker performance may differ among hyperandrogenic, ovulatory, lean, obese, and insulin-resistant phenotypes. Nevertheless, several candidate PCOS-specific modules have emerged. Increased BMP6 and reduced pentraxin 3 have been demonstrated in granulosa cells and FF from women with PCOS. BMP6 suppresses pentraxin 3 through ALK2/ALK3–SMAD1/5–SMAD4 signaling, while increased BMP6 expression has been associated with poorer oocyte maturation and embryo quality measures (93). FF GDF8 is also elevated in PCOS, and higher GDF8 accompanied by lower progesterone has been associated with poorer pregnancy outcomes (21).

PCOS FF additionally exhibits increased free fatty acids, 3-hydroxynonanoyl carnitine, deoxycorticosterone, phenylalanine, and leucine, together with reduced lysophosphatidylcholines and phytosphingosine (61). These findings indicate metabolic dysregulation but should be interpreted in relation to body mass index, insulin resistance, androgen concentrations, medication exposure, and PCOS phenotype. Although IL-6 may be increased in the PCOS follicular environment, IL-6 trans-signaling cannot be inferred from IL-6 alone. Assessment would require soluble IL-6 receptor-α and preferably soluble gp130 or downstream pathway activity. A provisional PCOS panel could therefore integrate BMP6/pentraxin 3, GDF8/progesterone, selected lipid and acylcarnitine metabolites, and IL-6/sIL-6Rα. Such a panel should first be validated for PCOS phenotype discrimination and subsequently for individual-oocyte and embryo outcomes (**Figure 4**).

PCOS-specific follicular-fluid panels are unlikely to have universally applicable reference ranges without validation. Candidate thresholds should be evaluated across well-defined PCOS phenotypes and diverse populations, with consideration of ancestry, body mass index, insulin resistance, androgen status, medication exposure, and stimulation protocol. External validation should determine whether a common model is transportable or whether phenotype- or population-specific recalibration is required.

## 9 Clinical translation: from bench to IVF practice

Translation of FF biomarker panels into IVF practice requires structured progression from biological plausibility to an analytically valid, clinically useful, and feasible test. The intended use and primary outcome, such as metaphase II oocyte maturity, normal fertilization, usable blastocyst formation, euploidy, implantation, or live birth, should first be clearly defined because these outcomes represent distinct biological stages. Candidate panels should integrate a limited number of complementary markers from biologically coherent domains, including periovulatory signaling, steroidogenesis, extracellular-matrix remodeling, metabolism, inflammation, oxidative stress, and mitochondrial function, rather than selecting analytes solely according to statistical significance. For follicle-level prediction, FF should be collected from individually aspirated follicles, with standardized handling of blood-contaminated or flushed samples, processing time, centrifugation, storage, and freeze–thaw exposure. Follicle size, stimulation regimen, trigger type, and trigger-to-retrieval interval should also be recorded, while preserving one-to-one linkage among FF, the corresponding oocyte, fertilization method, embryo, and subsequent outcome. Measurements from FF, mural granulosa cells, and cumulus cells should be distinguished because these compartments are not interchangeable.

Discovery-phase signatures should subsequently be reduced to parsimonious panels and converted into targeted assays with documented accuracy, precision, analytical sensitivity, linearity, recovery, stability, matrix effects, and interlaboratory reproducibility (114, 115). Predictive models should be developed using multilevel methods that account for multiple follicles within each woman, avoid information leakage during feature selection, and report discrimination, calibration, and incremental performance beyond age, ovarian reserve, follicle size, embryo morphology, and morphokinetics (106, 107).

Independent external validation across different populations, stimulation protocols, and laboratory platforms must precede prospective evaluation of whether biomarker-guided decisions improve clinically meaningful outcomes, including usable blastocyst formation, euploid embryo selection, time to pregnancy, and live birth (106, 107). Implementation will additionally depend on rapid turnaround, affordability, automation, compatibility with laboratory workflow, regulatory compliance, and transparent communication of uncertainty. Because FF is obtained only after follicular aspiration, its most plausible role is to complement rather than replace conventional embryological assessment. At present, no FF panel has demonstrated sufficient analytical validity, external reproducibility, and prospective clinical utility for routine IVF decision-making; therefore, the panels proposed in this review should remain investigational (**Table 2**).

## 10 Challenges, knowledge gaps, and future directions

Despite substantial progress in characterizing the follicular microenvironment, no FF biomarker or multianalyte panel has achieved sufficient analytical validity, clinical validity, and clinical utility for routine prediction of oocyte competence or IVF outcomes. Importantly, the candidate combinations proposed in this review are mechanism-informed, hypothesis-generating research frameworks rather than validated clinical panels. Many components were identified through experiments in cultured human granulosa-lutein cells, which are valuable for defining signaling relationships but do not reproduce the complete physiology of the developing follicle. Such studies cannot establish whether FF concentrations discriminate between competent and incompetent oocytes or provide predictive information beyond established clinical and embryological variables. Mechanistic plausibility, clinical association, and predictive utility should therefore be regarded as distinct levels of evidence. This distinction is particularly important because granulosa-lutein cell findings may not translate directly to the follicular compartment. Experimental changes in gene or protein expression do not necessarily produce corresponding changes in FF concentrations, and measurements obtained from mural granulosa cells, cumulus cells, and FF represent biologically distinct compartments. Moreover, the luteinized phenotype of cells collected after ovulation triggering may differ from the cellular state during earlier follicular development. Candidate pathways identified *in vitro* should therefore first be confirmed in more physiologically relevant systems, including primary cumulus–oocyte complexes, three-dimensional follicular cultures, organoids, and ex-vivo ovarian tissue. Subsequent clinical evaluation must determine whether the corresponding molecules are measurable in FF and associated with outcomes in individually matched follicle–oocyte–embryo datasets. The absence of such validation currently represents the principal limitation of the proposed panels.

Translation is further complicated by the dynamic biology and substantial heterogeneity of the follicle. FF provides a time-specific snapshot of interactions among the oocyte, granulosa and theca cells, vascular components, and immune cells. Its composition is influenced by follicle size, developmental stage, patient phenotype, gonadotropin exposure, trigger formulation, and the trigger-to-retrieval interval. Moreover, marked biochemical variation exists among follicles from the same woman (99). Pooling FF can obscure follicle-specific signals, whereas individual-follicle analysis creates clustered data that require multilevel statistical models. Treating multiple follicles or embryos from the same woman as independent observations underestimates uncertainty and increases the risk of false-positive associations.

Preanalytical and analytical heterogeneity further restricts reproducibility. Follicular flushing, blood contamination, processing delays, centrifugation, storage conditions, and freeze–thaw exposure can alter measured concentrations. Future studies should prioritize individually aspirated, unflushed, blood-free FF and preserve a one-to-one relationship among the sample, oocyte, and resulting embryo. Assays must also be evaluated for precision, recovery, linearity, sensitivity, matrix effects, analyte stability, and interlaboratory reproducibility (114). Discovery-phase omics candidates should be converted into targeted, clinically scalable assays before prospective evaluation. Existing evidence is predominantly derived from small, single-center, retrospective, or cross-sectional studies. Frequent methodological limitations include unrestricted feature selection, inadequate correction for multiple testing, performance assessment in the development dataset, and reporting of discrimination without calibration or clinical utility (105–107). Outcome heterogeneity is another major concern because oocyte maturity, fertilization, cleavage, embryo morphology, blastocyst development, euploidy, implantation, and live birth represent distinct biological stages. Future studies must define one primary outcome and adjust for important confounders, including age, ovarian reserve, body mass index, infertility diagnosis, PCOS phenotype, metabolic status, stimulation protocol, follicle size, trigger type, fertilization method, sperm quality, and embryology-laboratory practices.

A further gap is the disconnect between mechanistic and clinical evidence. Many proposed biomarkers regulate steroidogenesis, ECM remodeling, mitochondrial activity, or inflammatory signaling in cultured granulosa-lutein cells but have not demonstrated predictive value in matched follicle–oocyte cohorts. Mechanistic findings should therefore be confirmed in more physiologically relevant systems, including primary cumulus–oocyte complexes, three-dimensional follicular cultures, organoids, or ex-vivo ovarian tissue, and subsequently evaluated in individual-follicle clinical studies. Association alone should not be interpreted as evidence that modifying a biomarker will improve IVF outcomes. Future research should move from unrestricted biomarker discovery toward focused validation of biologically coherent, outcome-specific panels. Multicenter cohorts should integrate individually matched FF, cumulus or granulosa cells, oocytes, embryos, and live-birth follow-up. Multi-omics integration should be performed at the pathway level, with all preprocessing and feature selection restricted to training data. Models should undergo internal and blinded external validation and be assessed for discrimination, calibration, clinical utility, cost-effectiveness, and improvement over established clinical and embryological predictors (105–107). Phenotype-specific panels may be required for PCOS, endometriosis, diminished ovarian reserve, obesity, and reproductive aging. Single-cell, spatial, extracellular-vesicle, and longitudinal follicle-culture approaches may help identify the cellular origins and temporal behavior of candidate markers. Artificial intelligence may support multi-omics integration, but complex models should be adopted only when they provide reproducible advantages over simpler and more interpretable approaches (101, 102).

Ultimately, successful translation will depend not on progressively expanding biomarker signatures but on identifying the smallest analytically robust and biologically coherent panel that provides reproducible information beyond current clinical and embryological assessment. Until prospective, individually linked and externally validated evidence becomes available, the proposed FF biomarker combinations should remain investigational and should not guide patient treatment or embryo selection.

## 11 Conclusions

FF provides a biologically rich record of the endocrine, paracrine, metabolic, inflammatory, and extracellular-matrix processes that shape oocyte development. However, folliculogenesis is governed by interacting and temporally regulated signaling networks; consequently, no single FF analyte adequately captures oocyte competence or reliably predicts fertilization, blastocyst development, implantation, or live birth. Mechanistically informed panels integrating periovulatory growth factors, ECM components, steroid hormones, inflammatory mediators, oxidative-stress markers, mitochondrial indicators, and omics-derived metabolites are therefore more biologically plausible than isolated biomarkers (**Figure 5**).

**Figure 5:**
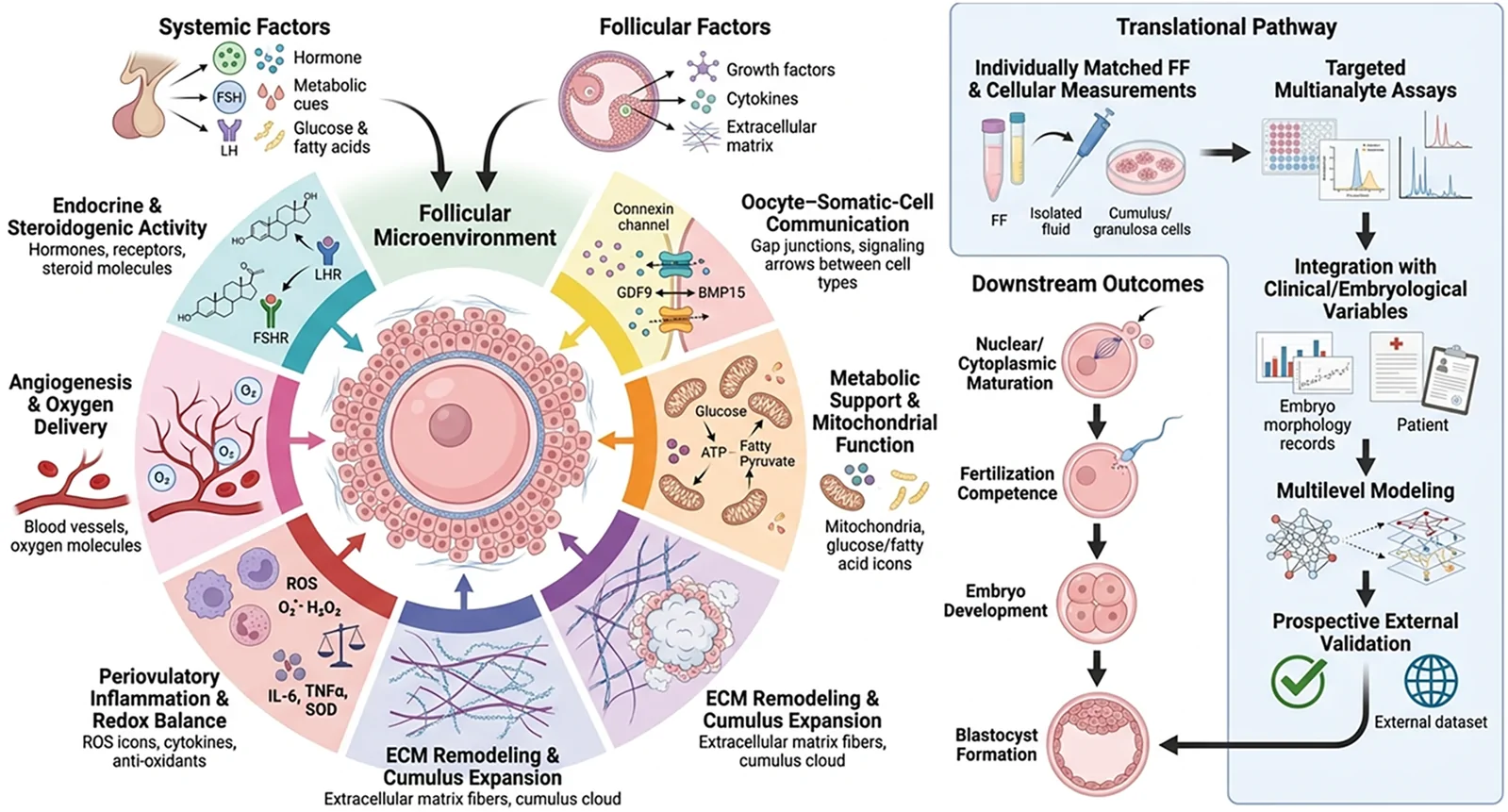
Integrative biological and translational framework linking the follicular microenvironment to oocyte competence and biomarker-panel development. Systemic inputs, including gonadotropins, hormones, metabolic cues, glucose, and fatty acids, interact with locally derived follicular factors, including growth factors, cytokines, and extracellular-matrix components, to shape the follicular microenvironment. These signals converge through interconnected biological domains comprising endocrine and steroidogenic activity, oocyte–somatic-cell communication, metabolic support and mitochondrial function, extracellular-matrix remodeling and cumulus expansion, periovulatory inflammation and redox balance, and angiogenesis and oxygen delivery. Together, these processes influence nuclear and cytoplasmic maturation, fertilization competence, embryo development, and blastocyst formation. The proposed translational pathway begins with individually matched follicular-fluid and cumulus or granulosa-cell measurements, followed by conversion of discovery signals into targeted multianalyte assays. Molecular measurements are integrated with patient characteristics and embryological variables using multilevel models that account for the clustering of follicles and embryos within patients. Prospective external validation is required before clinical implementation. The framework represents a proposed research and translation strategy rather than an established clinical test. ATP, adenosine triphosphate; BMP15, bone morphogenetic protein 15; ECM, extracellular matrix; FF, follicular fluid; FSH, follicle-stimulating hormone; FSHR, follicle-stimulating hormone receptor; GDF9, growth differentiation factor 9; H_2_O_2_, hydrogen peroxide; IL-6, interleukin-6; LH, luteinizing hormone; LHR, luteinizing hormone receptor; O_2_, molecular oxygen; ROS, reactive oxygen species; SOD, superoxide dismutase; TNF-α, tumor necrosis factor-α.

Among the candidates reviewed, AREG–pentraxin 3–PGE_2_ signaling, BMP6/pentraxin 3 and GDF8/progesterone balance, FF AMH and progesterone, redox and mitochondrial-injury markers, and selected metabolic signatures provide promising foundations for outcome- and phenotype-specific panels. Nevertheless, much of the supporting evidence derives from granulosa-cell experiments or small observational cohorts. Mechanistic activity should not be equated with clinical predictive utility, and PCOS-associated molecular signatures should not be generalized to all patients undergoing IVF.

Future development requires standardized individual-follicle sampling, one-to-one linkage of FF with the corresponding oocyte and embryo, targeted assay verification, multilevel statistical analysis, and independent prospective validation against clinically meaningful outcomes. Multi-omics integration may improve prediction when it is pathway-informed and adds reproducible information beyond established patient characteristics and embryological assessment. Until analytical validity, external reproducibility, clinical utility, feasibility, and cost-effectiveness are demonstrated, research frameworks for FF biomarkers should remain investigational. The ultimate objective is a small, interpretable, and clinically scalable panel that complements, not replaces, current embryological assessment and supports more precise IVF decision-making.

## Glossary

ALK2: activin receptor-like kinase 2
ALK3: activin receptor-like kinase 3
ALK4: activin receptor-like kinase 4
ALK5: activin receptor-like kinase 5
ALK6: activin receptor-like kinase 6
AMH: anti-Müllerian hormone
AREG: amphiregulin
BAMBI: BMP and activin membrane-bound inhibitor
BMP: bone morphogenetic protein
BMP2: bone morphogenetic protein 2
BMP4: bone morphogenetic protein 4
BMP6: bone morphogenetic protein 6
BMP7: bone morphogenetic protein 7
BMP15: bone morphogenetic protein 15
BNIP3: BCL2-interacting protein 3
BTC: betacellulin
COX-2: cyclooxygenase-2
CREB: cAMP response element-binding protein
CTGF: connective tissue growth factor
Cx37: connexin 37
Cx43: connexin 43
CXCL12: C-X-C motif chemokine ligand 12
CYP11A1: cytochrome P450 family 11 subfamily A member 1
CYP19A1: cytochrome P450 family 19 subfamily A member 1
EGF: epidermal growth factor
EGFR: epidermal growth factor receptor
ERBB1: erb-b2 receptor tyrosine kinase 1, also known as EGFR
EREG: epiregulin
ERK1/2: extracellular signal-regulated kinases 1 and 2
FSH: follicle-stimulating hormone
GDF8: growth differentiation factor 8
GDF9: growth differentiation factor 9
GDNF: glial cell line-derived neurotrophic factor
GFRA1: GDNF family receptor alpha 1
GJA1: gap junction protein alpha 1, encoding connexin 43
GJA4: gap junction protein alpha 4, encoding connexin 37
gp130: glycoprotein 130, also known as interleukin-6 signal transducer
HAS2: hyaluronan synthase 2
HIF: hypoxia-inducible factor
IGF-I: insulin-like growth factor I
IGF-II: insulin-like growth factor II
IGFBP-1–6: insulin-like growth factor-binding proteins 1–6
IL-6: interleukin-6
JAK2: Janus kinase 2
LC3: microtubule-associated protein 1 light chain 3
LOX: lysyl oxidase
miR-29a: microRNA-29a
miR-145-5p: microRNA-145-5p
MMP1: matrix metalloproteinase 1
PAPP-A: pregnancy-associated plasma protein A
PINK1: PTEN-induced kinase 1
PTGS2: prostaglandin-endoperoxide synthase 2, encoding COX-2
PTX3: pentraxin 3
RET: RET proto-oncogene receptor tyrosine kinase
SDF-1: stromal cell-derived factor 1, also known as CXCL12
sIL-6Rα: soluble interleukin-6 receptor alpha
SMAD1: SMAD family member 1
SMAD2: SMAD family member 2
SMAD3: SMAD family member 3
SMAD4: SMAD family member 4
SMAD5: SMAD family member 5
SMAD8: SMAD family member 8, also known as SMAD9
SNAIL: Snail family transcriptional repressor 1
SOCS3: suppressor of cytokine signaling 3
SOD: superoxide dismutase
SR-B1: scavenger receptor class B type 1
StAR: steroidogenic acute regulatory protein
STAT3: signal transducer and activator of transcription 3
TGF-β: transforming growth factor beta
TGF-β1: transforming growth factor beta 1
TNFα: tumor necrosis factor alpha
TNFAIP6: tumor necrosis factor alpha-induced protein 6
VCAN: versican
VEGF: vascular endothelial growth factor

## References

[1] Working Group on the update of the E.A.I.C Coticchio G Ahlstrom A Arroyo G Balaban B Campbell A . The Istanbul consensus update: a revised ESHRE/ALPHA consensus on oocyte and embryo static and dynamic morphological assessmentdagger,double dagger. Hum Reprod. (2025) 40:989–1035. doi: 10.1093/humrep/deaf021 PMC12127515 40288770

[2] Lie Fong S Baart EB Martini E Schipper I Visser JA Themmen AP . Anti-Mullerian hormone: a marker for oocyte quantity, oocyte quality and embryo quality? Reprod BioMed Online. (2008) 16:664–70. doi: 10.1016/s1472-6483(10)60480-4 18492370

[3] Chang HM Qiao J Leung PC . Oocyte-somatic cell interactions in the human ovary-novel role of bone morphogenetic proteins and growth differentiation factors. Hum Reprod Update. (2016) 23:1–18. doi: 10.1093/humupd/dmw039 27797914 PMC5155571

[4] Muraoka A Kajiyama H . Decoding the follicular fluid microenvironment: integrative biomarkers and multiomics signatures to predict IVF outcomes. Reprod Med Biol. (2026) 25:e70056. doi: 10.1002/rmb2.70056 42087984 PMC13137436

[5] Gilchrist RB Lane M Thompson JG . Oocyte-secreted factors: regulators of cumulus cell function and oocyte quality. Hum Reprod Update. (2008) 14:159–77. doi: 10.1093/humupd/dmm040 18175787

[6] Liu C Chang HM Yi Y Fang Y Zhao F Leung PCK . ALK4-SMAD2/3-SMAD4 signaling mediates the activin A-induced suppression of PTX3 in human granulosa-lutein cells. Mol Cell Endocrinol. (2019) 493:110485. doi: 10.1016/j.mce.2019.110485 31185247

[7] Tian S Zhang H Chang HM Klausen C Huang HF Jin M . Activin A promotes hyaluronan production and upregulates versican expression in human granulosa cellsdagger. Biol Reprod. (2022) 107:458–73. doi: 10.1093/biolre/ioac070 35403677 PMC9382401

[8] Alpha Scientists in Reproductive M E.S.I.G.o. Embryology . The Istanbul consensus workshop on embryo assessment: proceedings of an expert meeting. Hum Reprod. (2011) 26:1270–83. doi: 10.1093/humrep/der037 21502182

[9] Shalgi R Kraicer P Rimon A Pinto M Soferman N . Proteins of human follicular fluid: the blood-follicle barrier. Fertil Steril. (1973) 24:429–34. doi: 10.1016/s0015-0282(16)39730-8 4122802

[10] Fujimoto VY Kane JP Ishida BY Bloom MS Browne RW . High-density lipoprotein metabolism and the human embryo. Hum Reprod Update. (2010) 16:20–38. doi: 10.1093/humupd/dmp029 19700490

[11] Telfer EE Grosbois J Odey YL Rosario R Anderson RA . Making a good egg: human oocyte health, aging, and *in vitro* development. Physiol Rev. (2023) 103:2623–77. doi: 10.1152/physrev.00032.2022 37171807 PMC10625843

[12] Moolhuijsen LME Visser JA . Anti-mullerian hormone and ovarian reserve: update on assessing ovarian function. J Clin Endocrinol Metab. (2020) 105:3361–73. doi: 10.1210/clinem/dgaa513 32770239 PMC7486884

[13] Namwanje M Brown CW . Activins and inhibins: roles in development, physiology, and disease. Cold Spring Harb Perspect Biol. (2016) 8:a021881–935. doi: 10.1101/cshperspect.a021881 27328872 PMC4930927

[14] Hillier SG . Gonadotropic control of ovarian follicular growth and development. Mol Cell Endocrinol. (2001) 179:39–46. doi: 10.1016/s0303-7207(01)00469-5 11420129

[15] Dumesic DA Oberfield SE Stener-Victorin E Marshall JC Laven JS Legro RS . Scientific statement on the diagnostic criteria, epidemiology, pathophysiology, and molecular genetics of polycystic ovary syndrome. Endocr Rev. (2015) 36:487–525. doi: 10.1210/er.2015-1018 PMC4591526 26426951

[16] Agarwal A Gupta S Sharma RK . Role of oxidative stress in female reproduction. Reprod Biol Endocrinol. (2005) 3:28. doi: 10.1186/1477-7827-3-28 16018814 PMC1215514

[17] Russell DL Robker RL . Molecular mechanisms of ovulation: co-ordination through the cumulus complex. Hum Reprod Update. (2007) 13:289–312. doi: 10.1093/humupd/dml062 17242016

[18] Chang H-M Cheng JC Fang L Qiu X Klausen C Taylor EL . Recombinant BMP4 and BMP7 downregulate pentraxin 3 in human granulosa cells. J Clin Endocrinol Metab. (2015) 100:E365–74. doi: 10.1210/jc.2014-2496 25514099

[19] Zheng X Zheng Y Qin D Yao Y Zhang X Zhao Y . Regulatory role and potential importance of GDF-8 in ovarian reproductive activity. Front Endocrinol (Lausanne). (2022) 13:878069. doi: 10.3389/fendo.2022.878069 35692411 PMC9178251

[20] Fang L Yu Y Zhang R He J Sun Y-P . Serum GDF-8 levels change dynamically during controlled ovarian hyperstimulation in patients undergoing IVF/ICSI-ET. Sci Rep. (2016) 6:28036. doi: 10.1038/srep28036 27329327 PMC4916440

[21] Fang L Wang S Li Y Yu Y Li Y Yan Y . High GDF-8 in follicular fluid is associated with a low pregnancy rate in IVF patients with PCOS. Reproduction. (2020) 160:11–9. doi: 10.1530/rep-20-0077 32272446

[22] Chang HM Cheng JC Klausen C Leung PC . Recombinant BMP4 and BMP7 increase activin A production by up-regulating inhibin βA subunit and furin expression in human granulosa-lutein cells. J Clin Endocrinol Metab. (2015) 100:E375–86. doi: 10.1210/jc.2014-3026 25562508

[23] Tian S Zhang H Chang H Klausen C Huang H Jin M . Activin A promotes hyaluronan production and upregulates versican expression in human granulosa cells†. Biol Reprod. (2022) 107:458–73. doi: 10.1093/biolre/ioac070 35403677 PMC9382401

[24] Chang HM Bai L Zhu YM Leung PCK . Connective tissue growth factor mediates bone morphogenetic protein 2-induced increase in hyaluronan production in luteinized human granulosa cells. Reprod Biol Endocrinol. (2022) 20:65. doi: 10.1186/s12958-022-00937-y 35395768 PMC8991488

[25] Bongrani A Plotton I Mellouk N Rame C Guerif F Froment P . High androgen concentrations in follicular fluid of polycystic ovary syndrome women. Reprod Biol Endocrinol. (2022) 20:88. doi: 10.1186/s12958-022-00959-6 35701786 PMC9195430

[26] Teissier MP Chable H Paulhac S Aubard Y . Comparison of follicle steroidogenesis from normal and polycystic ovaries in women undergoing IVF: relationship between steroid concentrations, follicle size, oocyte quality and fecundability. Hum Reprod. (2000) 15:2471–7. doi: 10.1093/humrep/15.12.2471 11098013

[27] Cui L Fang L Mao X Chang HM Leung PCK Ye Y . GDNF-induced downregulation of miR-145-5p enhances human oocyte maturation and cumulus cell viability. J Clin Endocrinol Metab. (2018) 103:2510–21. doi: 10.1210/jc.2017-02742 29897461

[28] Robinson RS Woad KJ Hammond AJ Laird M Hunter MG Mann GE . Angiogenesis and vascular function in the ovary. Reproduction. (2009) 138:869–81. doi: 10.1530/rep-09-0283 19786399

[29] Artini PG Ruggiero M Monteleone P Carpi A Cristello F Cela V . Vascular endothelial growth factor and its soluble receptor in benign and malignant ovarian tumors. BioMed Pharmacother. (2008) 62:373–7. doi: 10.1016/j.biopha.2007.10.005 18037256

[30] Nishigaki A Tsuzuki-Nakao T Kido T Kida N Kakita-Kobayashi M Murata H . Concentration of stromal cell-derived factor-1 (SDF-1/CXCL12) in the follicular fluid is associated with blastocyst development. Reprod Med Biol. (2019) 18:161–6. doi: 10.1002/rmb2.12259 PMC6452007 30996679

[31] Fried G Wramsby H Tally M . Transforming growth factor-beta1, insulin-like growth factors, and insulin-like growth factor binding proteins in ovarian follicular fluid are differentially regulated by the type of ovarian hyperstimulation used for *in vitro* fertilization. Fertil Steril. (1998) 70:129–34. doi: 10.1016/s0015-0282(98)00105-8 9660434

[32] Albeitawi S Bani-Mousa SU Jarrar B Aloqaily I Al-Shlool N Alsheyab G . Associations between follicular fluid biomarkers and IVF/ICSI outcomes in normo-ovulatory women-a systematic review. Biomolecules. (2025) 15:443–82. doi: 10.3390/biom15030443 40149979 PMC11940193

[33] Bracewell-Milnes T Saso S Abdalla H Nikolau D Norman-Taylor J Johnson M . Metabolomics as a tool to identify biomarkers to predict and improve outcomes in reproductive medicine: a systematic review. Hum Reprod Update. (2017) 23:723–36. doi: 10.1093/humupd/dmx023 29069503

[34] Zhang Y Xia X Yan J Yan L Lu C Zhu X . Mesenchymal stem cell-derived angiogenin promotes primodial follicle survival and angiogenesis in transplanted human ovarian tissue. Reprod Biol Endocrinol. (2017) 15:18. doi: 10.1186/s12958-017-0235-8 28274269 PMC5343383

[35] Dumesic DA Meldrum DR Katz-Jaffe MG Krisher RL Schoolcraft WB . Oocyte environment: follicular fluid and cumulus cells are critical for oocyte health. Fertil Steril. (2015) 103:303–16. doi: 10.1016/j.fertnstert.2014.11.015 25497448

[36] Revelli A Delle Piane L Casano S Molinari E Massobrio M Rinaudo P . Follicular fluid content and oocyte quality: from single biochemical markers to metabolomics. Reprod Biol Endocrinol. (2009) 7:40. doi: 10.1186/1477-7827-7-40 19413899 PMC2685803

[37] Nagy B Poto L Farkas N Koppan M Varnagy A Kovacs K . Follicular fluid progesterone concentration is associated with fertilization outcome after IVF: a systematic review and meta-analysis. Reprod BioMed Online. (2019) 38:871–82. doi: 10.1016/j.rbmo.2018.12.045 30948305

[38] Manau D Balasch J Jimenez W Fabregues F Civico S Casamitjana R . Follicular fluid concentrations of adrenomedullin, vascular endothelial growth factor and nitric oxide in IVF cycles: relationship to ovarian response. Hum Reprod. (2000) 15:1295–9. doi: 10.1093/humrep/15.6.1295 10831558

[39] Barroso G Barrionuevo M Rao P Graham L Danforth D Huey S . Vascular endothelial growth factor, nitric oxide, and leptin follicular fluid levels correlate negatively with embryo quality in IVF patients. Fertil Steril. (1999) 72:1024–6. doi: 10.1016/s0015-0282(99)00442-2 10593375

[40] Inoue Y Miyamoto S Fukami T Shirota K Yotsumoto F Kawarabayashi T . Amphiregulin is much more abundantly expressed than transforming growth factor-alpha and epidermal growth factor in human follicular fluid obtained from patients undergoing *in vitro* fertilization-embryo transfer. Fertil Steril. (2009) 91:1035–41. doi: 10.1016/j.fertnstert.2008.01.014 18325497

[41] Kidder GM Vanderhyden BC . Bidirectional communication between oocytes and follicle cells: ensuring oocyte developmental competence. Can J Physiol Pharmacol. (2010) 88:399–413. doi: 10.1139/y10-009 20555408 PMC3025001

[42] Kidder GM Mhawi AA . Gap junctions and ovarian folliculogenesis. Reproduction. (2002) 123:613–20. doi: 10.1530/rep.0.1230613 12006089

[43] Chen YC Chang HM Cheng JC Tsai HD Wu CH Leung PC . Transforming growth factor-beta1 up-regulates connexin43 expression in human granulosa cells. Hum Reprod. (2015) 30:2190–201. doi: 10.1093/humrep/dev175 26202915 PMC4542719

[44] Fang L Chang H-M Cheng J-C Leung PCK Sun Y-P . TGF-β1 downregulates stAR expression and decreases progesterone production through smad3 and ERK1/2 signaling pathways in human granulosa cells. J Clin Endocrinol Metab. (2014) 99:E2234–43. doi: 10.1210/jc.2014-1930 25140399

[45] Chang H Cheng J Huang H Shi F Leung P . Activin A, B and AB decrease progesterone production by down-regulating StAR in human granulosa cells. Mol Cell Endocrinol. (2015) 412:290–301. doi: 10.1016/j.mce.2015.05.016 26001835

[46] Fang L Chang H Cheng J Yu Y Leung P Sun Y . Growth differentiation factor-8 decreases stAR expression through ALK5-mediated smad3 and ERK1/2 signaling pathways in luteinized human granulosa cells. Endocrinology. (2015) 156:4684–94. doi: 10.1016/j.fertnstert.2015.07.330 26393302

[47] Chang H Klausen C Leung P . Antimüllerian hormone inhibits follicle-stimulating hormone-induced adenylyl cyclase activation, aromatase expression, and estradiol production in human granulosa-lutein cells'. Fertility Sterility. (2025) 124:796. doi: 10.1016/j.fertnstert.2013.04.019 23663993

[48] Fang L Cheng J Chang H Sun Y Leung P . EGF-like growth factors induce COX-2-derived PGE2 production through ERK1/2 in human granulosa cells. J Clin Endocrinol Metab. (2013) 98:4932–41. doi: 10.1210/jc.2013-2662 24092824

[49] Fang L Chang HM Cheng JC Leung PC Sun YP . Nitric oxide and cGMP induce COX-2 expression and PGE2 production in human granulosa cells through CREB signaling pathway. J Clin Endocrinol Metab. (2015) 100:E262–9. doi: 10.1210/jc.2014-2886 25423567

[50] Li SJ Chang HM Xie J Wang JH Yang J Leung PCK . The interleukin 6 trans-signaling increases prostaglandin E2 production in human granulosa cellsdagger. Biol Reprod. (2021) 105:1189–204. doi: 10.1093/biolre/ioab128 PMC8599049 34198336

[51] Kedem-Dickman A Maman E Yung Y Yerushalmi GM Hemi R Hanochi M . Anti-Müllerian hormone is highly expressed and secreted from cumulus granulosa cells of stimulated preovulatory immature and atretic oocytes. Reprod BioMedicine Online. (2012) 24:540–6. doi: 10.1016/j.rbmo.2012.01.023 22421733

[52] Naessen T Kushnir MM Chaika A Nosenko J Mogilevkina I Rockwood AL . Steroid profiles in ovarian follicular fluid in women with and without polycystic ovary syndrome, analyzed by liquid chromatography-tandem mass spectrometry. Fertil Steril. (2010) 94:2228–33. doi: 10.1016/j.fertnstert.2009.12.081 20171618

[53] Carpintero NL Suarez OA Varea CG Rioja RG Mangas CC . Variability between the follicular steroid hormone levels in different follicles of the same patient and between patients. J Hum Reprod Sci. (2015) 8:37–42. doi: 10.4103/0974-1208.153125 25838747 PMC4381381

[54] Monteleone P Giovanni Artini P Simi G Casarosa E Cela V Genazzani AR . Follicular fluid VEGF levels directly correlate with perifollicular blood flow in normoresponder patients undergoing IVF. J Assist Reprod Genet. (2008) 25:183–6. doi: 10.1007/s10815-008-9218-1 18449636 PMC2582064

[55] Fried G Remaeus K Harlin J Krog E Csemiczky G Aanesen A . Inhibin B predicts oocyte number and the ratio IGF-I/IGFBP-1 may indicate oocyte quality during ovarian hyperstimulation for *in vitro* fertilization. J Assist Reprod Genet. (2003) 20:167–76. doi: 10.1023/a:1023656225053 12812459 PMC3455298

[56] Dai Y Wai S Li P Shan N Cao Z Li Y . Soft hydrogel semiconductors with augmented biointeractive functions. Science. (2024) 386:431–9. doi: 10.1126/science.adp9314 39446940 PMC12392907

[57] Scalici E Traver S Molinari N Mullet T Monforte M Vintejoux E . Cell-free DNA in human follicular fluid as a biomarker of embryo quality. Hum Reprod. (2014) 29:2661–9. doi: 10.1093/humrep/deu238 25267787

[58] Schenk M Huppertz B Obermayer-Pietsch B Kastelic D Hormann-Kropfl M Weiss G . Biobanking of different body fluids within the frame of IVF-a standard operating procedure to improve reproductive biology research. J Assist Reprod Genet. (2017) 34:283–90. doi: 10.1007/s10815-016-0847-5 PMC5306411 27889868

[59] Yland J Messerlian C Minguez-Alarcon L Ford JB Hauser R Williams PL . Methodological approaches to analyzing IVF data with multiple cycles. Hum Reprod. (2019) 34:549–57. doi: 10.1093/humrep/dey374 PMC6389861 30576499

[60] Ren Q Wang YL Wang ML Wang HY . Screening and identification of the metabolites in rat urine and feces after oral administration of Lycopus lucidus Turcz extract by UHPLC-Q-TOF-MS mass spectrometry. J Chromatogr B Analyt Technol BioMed Life Sci. (2016) 1027:64–73. doi: 10.1016/j.jchromb.2016.05.020 27262082

[61] Sun Z Chang H-M Wang A Song J Zhang X Guo J . Identification of potential metabolic biomarkers of polycystic ovary syndrome in follicular fluid by SWATH mass spectrometry. Reprod Biol Endocrinol. (2019) 17:45. doi: 10.1186/s12958-019-0490-y 31186025 PMC6560878

[62] Sun Z Song J Zhang X Wang A Guo Y Yang Y . SWATHHM-based metabolomics of follicular fluid in patients shows that progesterone adversely affects oocyte quality. BioMed Res Int. (2018) 2018:1780391. doi: 10.1155/2018/1780391 29951527 PMC5987297

[63] D'Arco Correa L Souza Peruzzato F Rauh Muller YM . Metabolomic alterations in follicular fluid across female infertility conditions in IVF: a systematic review. J Assist Reprod Genet. (2026) 43:2321–39. doi: 10.1007/s10815-026-03925-y 42237000 PMC13597975

[64] Cadenas J Poulsen LC Nikiforov D Grondahl ML Kumar A Bahnu K . Regulation of human oocyte maturation *in vivo* during the final maturation of follicles. Hum Reprod. (2023) 38:686–700. doi: 10.1093/humrep/dead024 36762771

[65] Li Y Chang HM Zhu H Sun YP Leung PCK . EGF-like growth factors upregulate pentraxin 3 expression in human granulosa-lutein cells. J Ovarian Res. (2024) 17:97. doi: 10.1186/s13048-024-01404-5 38720330 PMC11077866

[66] Paffoni A Ragni G Doni A Somigliana E Pasqualini F Restelli L . Follicular fluid levels of the long pentraxin PTX3. J Soc Gynecol Investig. (2006) 13:226–31. doi: 10.1016/j.jsgi.2005.12.008 16516502

[67] Chang HM Fang L Cheng JC Klausen C Sun YP Leung PC . Growth differentiation factor 8 down-regulates pentraxin 3 in human granulosa cells. Mol Cell Endocrinol. (2015) 404:82–90. doi: 10.1016/j.mce.2015.01.036 25641196

[68] Li H Chang HM Shi Z Leung PCK . SNAIL mediates TGF-beta1-induced downregulation of pentraxin 3 expression in human granulosa cells. Endocrinology. (2018) 159:1644–57. doi: 10.1210/en.2017-03127 29462303 PMC5939639

[69] Zhang X Jafari N Barnes RB Confino E Milad M Kazer RR . Studies of gene expression in human cumulus cells indicate pentraxin 3 as a possible marker for oocyte quality. Fertil Steril. (2005) 83 Suppl 1:1169–79. doi: 10.1016/j.fertnstert.2004.11.030 15831290

[70] Cillo F Brevini TA Antonini S Paffoni A Ragni G Gandolfi F . Association between human oocyte developmental competence and expression levels of some cumulus genes. Reproduction. (2007) 134:645–50. doi: 10.1530/rep-07-0182 17965254

[71] Chang HM Cheng JC Klausen C Leung PC . Recombinant BMP4 and BMP7 increase activin A production by up-regulating inhibin betaA subunit and furin expression in human granulosa-lutein cells. J Clin Endocrinol Metab. (2015) 100:E375–86. doi: 10.1210/jc.2014-3026 25562508

[72] Zhang H Klausen C Zhu H Chang HM Leung PC . BMP4 and BMP7 suppress StAR and progesterone production via ALK3 and SMAD1/5/8-SMAD4 in human granulosa-lutein cells. Endocrinology. (2015) 11:4269–80. doi: 10.1210/en.2015-1494 26302112

[73] Liu S Chang HM Yi Y Yao YQ Leung PCK . SMAD-dependent signaling mediates morphogenetic protein 6-induced stimulation of connective tissue growth factor in luteinized human granulosa cells†. Biol Reprod. (2019) 101:445–56. doi: 10.1093/biolre/ioz108 31210269 PMC6736146

[74] Yang J Li Y Li S Zhang Y Feng R Huang R . Metabolic signatures in human follicular fluid identify lysophosphatidylcholine as a predictor of follicular development. Commun Biol. (2022) 5:763. doi: 10.1038/s42003-022-03710-4 35906399 PMC9334733

[75] Youle RJ van der Bliek AM . Mitochondrial fission, fusion, and stress. Science. (2012) 337:1062–5. doi: 10.1126/science.1219855 22936770 PMC4762028

[76] Wu HC Chang HM Yi Y Sun ZG Lin YM Lian F . Bone morphogenetic protein 6 affects cell-cell communication by altering the expression of Connexin43 in human granulosa-lutein cells. Mol Cell Endocrinol. (2019) 498:110548. doi: 10.1016/j.mce.2019.110548 31434001

[77] May-Panloup P Boucret L Chao de la Barca JM Desquiret-Dumas V Ferre-L'Hotellier V Moriniere C . Ovarian ageing: the role of mitochondria in oocytes and follicles. Hum Reprod Update. (2016) 22:725–43. doi: 10.1093/humupd/dmw028 27562289

[78] Chang H-M Cheng J-C Liu Y Klausen C Xu C Leung PCK . Activin A-induced increase in LOX activity in human granulosa–lutein cells is mediated by CTGF. Reproduction. (2016) 152:293–301. doi: 10.1530/rep-16-0254 27530347

[79] Cataldo NA Rabinovici J Fujimoto VY Jaffe RB . Follistatin antagonizes the effects of activin-A on steroidogenesis in human luteinizing granulosa cells. J Clin Endocrinol Metab. (1994) 79:272–7. doi: 10.1210/jc.79.1.272 7517947

[80] Oyawoye O Abdel Gadir A Garner A Constantinovici N Perrett C Hardiman P . Antioxidants and reactive oxygen species in follicular fluid of women undergoing IVF: relationship to outcome. Hum Reprod. (2003) 18:2270–4. doi: 10.1093/humrep/deg450 14585872

[81] Nishihara T Matsumoto K Hosoi Y Morimoto Y . Evaluation of antioxidant status and oxidative stress markers in follicular fluid for human *in vitro* fertilization outcome. Reprod Med Biol. (2018) 17:481–6. doi: 10.1002/rmb2.12229 30377403 PMC6194301

[82] Collado-Fernandez E Picton HM Dumollard R . Metabolism throughout follicle and oocyte development in mammals. Int J Dev Biol. (2012) 56:799–808. doi: 10.1387/ijdb.120140ec 23417402

[83] Palikaras K Lionaki E Tavernarakis N . Mechanisms of mitophagy in cellular homeostasis, physiology and pathology. Nat Cell Biol. (2018) 20:1013–22. doi: 10.1038/s41556-018-0176-2 30154567

[84] Desquiret-Dumas V Clement A Seegers V Boucret L Ferre-L'Hotellier V Bouet PE . The mitochondrial DNA content of cumulus granulosa cells is linked to embryo quality. Hum Reprod. (2017) 32:607–14. doi: 10.1093/humrep/dew341 28077604

[85] Ventayol-Guirado M Hernandez-Rodriguez J Florit J Llull-Alberti MV Barragan R Ferragut JF . First follicular fluid cf-mtDNA sequencing unveils ovarian stimulation-induced mutations impacting oocyte quality and IVF success. J Assist Reprod Genet. (2025) 42:2375–86. doi: 10.1007/s10815-025-03511-8 40405035 PMC13633218

[86] Kazemi A Ramezanzadeh F Nasr-Esfahani MH Saboor Yaraghi AA Ahmadi M . Does dietary fat intake influence oocyte competence and embryo quality by inducing oxidative stress in follicular fluid? Iran J Reprod Med. (2013) 11:1005–12. PMC3941410 24639727

[87] Jana SK K NB Chattopadhyay R Chakravarty B Chaudhury K . Upper control limit of reactive oxygen species in follicular fluid beyond which viable embryo formation is not favorable. Reprod Toxicol. (2010) 29:447–51. doi: 10.1016/j.reprotox.2010.04.002 20406678

[88] Richards JS Ascoli M . Endocrine, paracrine, and autocrine signaling pathways that regulate ovulation. Trends Endocrinol Metab. (2018) 29:313–25. doi: 10.1016/j.tem.2018.02.012 29602523

[89] Duffy DM Ko C Jo M Brannstrom M Curry TE . Ovulation: parallels with inflammatory processes. Endocr Rev. (2019) 40:369–416. doi: 10.1210/er.2018-00075 PMC6405411 30496379

[90] Altun T Jindal S Greenseid K Shu J Pal L . Low follicular fluid IL-6 levels in IVF patients are associated with increased likelihood of clinical pregnancy. J Assist Reprod Genet. (2011) 28:245–51. doi: 10.1007/s10815-010-9502-8 PMC3082657 21046223

[91] Yang J Yang X Yang H Bai Y Zha H Jiang F . Interleukin 6 in follicular fluid reduces embryo fragmentation and improves the clinical pregnancy rate. J Assist Reprod Genet. (2020) 37:1171–6. doi: 10.1007/s10815-020-01737-2 32189182 PMC7244683

[92] Pierre A Peigne M Grynberg M Arouche N Taieb J Hesters L . Loss of LH-induced down-regulation of anti-Mullerian hormone receptor expression may contribute to anovulation in women with polycystic ovary syndrome. Hum Reprod. (2013) 28:762–9. doi: 10.1093/humrep/des460 23321213

[93] Xin X Chang HM Leung PCK Dong L Li J Lian F . Bone morphogenetic protein 6 induces downregulation of pentraxin 3 expression in human granulosa lutein cells in women with polycystic ovary syndrome. J Assist Reprod Genet. (2024) 41:31–48. doi: 10.1007/s10815-023-02972-z PMC10789681 37930517

[94] Zhang XY Chang HM Zhu H Liu RZ Leung PCK . BMP6 increases TGF-beta1 production by up-regulating furin expression in human granulosa-lutein cells. Cell Signal. (2019) 55:109–18. doi: 10.1016/j.cellsig.2019.01.002 30633987

[95] Gillet LC Navarro P Tate S Rost H Selevsek N Reiter L . Targeted data extraction of the MS/MS spectra generated by data-independent acquisition: a new concept for consistent and accurate proteome analysis. Mol Cell Proteomics. (2012) 11:O111 016717. doi: 10.1074/mcp.o111.016717 22261725 PMC3433915

[96] Collins BC Hunter CL Liu Y Schilling B Rosenberger G Bader SL . Multi-laboratory assessment of reproducibility, qualitative and quantitative performance of SWATH-mass spectrometry. Nat Commun. (2017) 8:291. doi: 10.1038/s41467-017-00249-5 28827567 PMC5566333

[97] Ambekar AS Nirujogi RS Srikanth SM Chavan S Kelkar DS Hinduja I . Proteomic analysis of human follicular fluid: a new perspective towards understanding folliculogenesis. J Proteomics. (2013) 87:68–77. doi: 10.1016/j.jprot.2013.05.017 23707233

[98] Zamah AM Hassis ME Albertolle ME Williams KE . Proteomic analysis of human follicular fluid from fertile women. Clin Proteomics. (2015) 12:5. doi: 10.1186/s12014-015-9077-6 25838815 PMC4357057

[99] Lewandowska AE Macur K Czaplewska P Liss J Lukaszuk K Oldziej S . Qualitative and quantitative analysis of proteome and peptidome of human follicular fluid using multiple samples from single donor with LC-MS and SWATH methodology. J Proteome Res. (2017) 16:3053–67. doi: 10.1021/acs.jproteome.7b00366 28658951

[100] Fang Y Chang HM Cheng JC Klausen C Leung PC Yang X . Transforming growth factor-beta1 increases lysyl oxidase expression by downregulating MIR29A in human granulosa lutein cells. Reproduction. (2016) 152:205–13. doi: 10.1530/rep-16-0144 27335131

[101] Hasin Y Seldin M Lusis A . Multi-omics approaches to disease. Genome Biol. (2017) 18:83. doi: 10.1186/s13059-017-1215-1 28476144 PMC5418815

[102] Karczewski KJ Snyder MP . Integrative omics for health and disease. Nat Rev Genet. (2018) 19:299–310. doi: 10.1038/nrg.2018.4 29479082 PMC5990367

[103] Chen Y Xie M Wu S Deng Z Tang Y Guan Y . Multi-omics approach to reveal follicular metabolic changes and their effects on oocyte competence in PCOS patients. Front Endocrinol (Lausanne). (2024) 15:1426517. doi: 10.3389/fendo.2024.1426517 39464191 PMC11502346

[104] Simon R Radmacher MD Dobbin K McShane LM . Pitfalls in the use of DNA microarray data for diagnostic and prognostic classification. J Natl Cancer Inst. (2003) 95:14–8. doi: 10.1093/jnci/95.1.14 12509396

[105] Castaldi PJ Dahabreh IJ Ioannidis JP . An empirical assessment of validation practices for molecular classifiers. Brief Bioinform. (2011) 12:189–202. doi: 10.1093/bib/bbq073 21300697 PMC3088312

[106] Collins GS Reitsma JB Altman DG Moons KG . Transparent reporting of a multivariable prediction model for individual prognosis or diagnosis (TRIPOD): the TRIPOD statement. Ann Intern Med. (2015) 162:55–63. doi: 10.1186/s12916-014-0241-z 25560714

[107] Wolff RF Moons KGM Riley RD Whiting PF Westwood M Collins GS . PROBAST: a tool to assess the risk of bias and applicability of prediction model studies. Ann Intern Med. (2019) 170:51–8. doi: 10.7326/m18-1376 30596875

[108] Chang HM Cheng JC Liu Y Klausen C Xu C Leung PC . Activin A-induced increase in LOX activity in human granulosa-lutein cells is mediated by CTGF. Reproduction. (2016) 152:293–301. doi: 10.1530/rep-16-0254 27530347

[109] Kristensen SG Kumar A Kalra B Pors SE Botkjaer JA Mamsen LS . Quantitative differences in TGF-beta family members measured in small antral follicle fluids from women with or without PCO. J Clin Endocrinol Metab. (2019) 104:6371–84. doi: 10.1210/jc.2019-01094 31287539

[110] Li H Chang HM Shi Z Leung PCK . ID3 mediates the TGF-beta1-induced suppression of matrix metalloproteinase-1 in human granulosa cells. FEBS J. (2019) 286:4310–27. doi: 10.1111/febs.14964 31215762

[111] Kim JH Lee JR Chang HJ Jee BC Suh CS Kim SH . Anti-Mullerian hormone levels in the follicular fluid of the preovulatory follicle: a predictor for oocyte fertilization and quality of embryo. J Korean Med Sci. (2014) 29:1266–70. doi: 10.3346/jkms.2014.29.9.1266 25246746 PMC4168181

[112] Ciepiela P Duleba AJ Kario A Chelstowski K Branecka-Wozniak D Kurzawa R . Oocyte matched follicular fluid anti-Mullerian hormone is an excellent predictor of live birth after fresh single embryo transfer. Hum Reprod. (2019) 34:2244–53. doi: 10.1093/humrep/dez186 31725884

[113] O'Brien Y Wingfield M O'Shea LC . Anti-Mullerian hormone and progesterone levels in human follicular fluid are predictors of embryonic development. Reprod Biol Endocrinol. (2019) 17:47. doi: 10.1186/s12958-019-0492-9 PMC6585091 31217014

[114] McShane LM Cavenagh MM Lively TG Eberhard DA Bigbee WL Williams PM . Criteria for the use of omics-based predictors in clinical trials. Nature. (2013) 502:317–20. doi: 10.1038/nature12564 24132288 PMC4180668

[115] Hayes DF . Biomarker validation and testing. Mol Oncol. (2015) 9:960–6. doi: 10.1016/j.molonc.2014.10.004 PMC5528748 25458054

